# Genome-Wide Screen for *Saccharomyces cerevisiae* Genes Contributing to Opportunistic Pathogenicity in an Invertebrate Model Host

**DOI:** 10.1534/g3.117.300245

**Published:** 2017-11-09

**Authors:** Sujal S. Phadke, Calum J. Maclean, Serena Y. Zhao, Emmi A. Mueller, Lucas A. Michelotti, Kaitlyn L. Norman, Anuj Kumar, Timothy Y. James

**Affiliations:** * Department of Ecology and Evolutionary Biology, University of Michigan, Ann Arbor, Michigan 48109; † Department of Molecular, Cellular, and Developmental Biology, University of Michigan, Ann Arbor, Michigan 48109

**Keywords:** virulence, barseq, pseudohyphae, hybrid, bulk segregant analysis

## Abstract

Environmental opportunistic pathogens can exploit vulnerable hosts through expression of traits selected for in their natural environments. Pathogenicity is itself a complicated trait underpinned by multiple complex traits, such as thermotolerance, morphology, and stress response. The baker’s yeast, *Saccharomyces cerevisiae*, is a species with broad environmental tolerance that has been increasingly reported as an opportunistic pathogen of humans. Here we leveraged the genetic resources available in yeast and a model insect species, the greater waxmoth *Galleria mellonella*, to provide a genome-wide analysis of pathogenicity factors. Using serial passaging experiments of genetically marked wild-type strains, a hybrid strain was identified as the most fit genotype across all replicates. To dissect the genetic basis for pathogenicity in the hybrid isolate, bulk segregant analysis was performed which revealed eight quantitative trait loci significantly differing between the two bulks with alleles from both parents contributing to pathogenicity. A second passaging experiment with a library of deletion mutants for most yeast genes identified a large number of mutations whose relative fitness differed *in vivo* *vs.* *in vitro*, including mutations in genes controlling cell wall integrity, mitochondrial function, and tyrosine metabolism. Yeast is presumably subjected to a massive assault by the innate insect immune system that leads to melanization of the host and to a large bottleneck in yeast population size. Our data support that resistance to the innate immune response of the insect is key to survival in the host and identifies shared genetic mechanisms between *S. cerevisiae* and other opportunistic fungal pathogens.

Understanding the evolution of pathogenicity (ability to infect a host) and virulence (degree of host damage) is critical to understanding the biology of nearly half of all life forms (Dobson *et al.* 2008) and managing the spread and treatment of infectious diseases. Natural selection on pathogenicity and virulence traits vary greatly depending on the parasite life cycle. For example, opportunistic pathogens—those that cause damage to a host dependent upon environment and host condition (Casadevall and Pirofski 1999)—include those whose primary niche is outside of the host, such as saprobic fungi. The observation of variable pathogenicity across fungi is enigmatic, nonetheless, the coincidental selection or accidental virulence hypotheses can account for the virulence of environmental opportunistic pathogens as a by-product of selection for traits in their natural habitats (Casadevall and Pirofski 2007; Ebert and Bull 2008). Despite the accidental nature of the virulence, insights into what traits are pathogenicity factors in environmental opportunistic pathogens can be obtained using genetic and comparative approaches.

Fungi represent a large subset of all pathogens, with many species causing opportunistic infections in animals including humans. Consequently, the genetic machinery underlying pathogenicity has been actively studied in many fungi of which species of *Candida*, *Cryptococcus*, and *Aspergillus* have been the leading models due to the high number of infection cases associated with them (Odds 2000; Richardson and Lass-Flörl 2008). These studies show that opportunistic fungal pathogens of humans possess a set of factors that allow them to function as pathogens. Pathogenicity or virulence factors for fungi causing mycoses are known to include: the ability to grow above 37°, adhesion, cell wall integrity, melanin formation, iron scavenging, and mitochondrial function to name a few (van Burik and Magee 2001; Alspaugh 2015; Calderone *et al.* 2015). Morphological factors are also important: hypha and pseudohyphal forms are associated with penetration, yeast forms with dissemination and migration, and extraordinary large forms, so-called titan cells, with macrophage evasion (Zaragoza and Nielsen 2013). Major insights into the pathogenicity of environmental opportunistic fungi followed from the appreciation that some of the pathogenicity factors are likely the product of selection to avoid predation by amoebae and nematodes in the wild (Casadevall *et al.* 2003).

Most insights into the genetics of opportunistic fungi have been generated by using the leading clinical fungal species. However, these systems, while biologically relevant, are not ideal model systems for genetic investigation. For example, *Candida albicans* does not undergo a standard sexual cycle, and targeted genetic transformation is challenging in both *Aspergillus* and *Cryptococcus*. Moreover, because there is known variation in virulence across isolates of a single species (Fraser *et al.* 2005; Kowalski *et al.* 2016), leveraging this genetic variation would provide a major source of natural variation to determine the mechanisms and evolution of pathogenicity in opportunistic fungi. In this study, we leveraged the power and genetic variation of a leading model fungus, *Saccharomyces cerevisiae*, to address whether its genetic resources can be used to identify genes of relevance to virulence in opportunistic fungi.

*S. cerevisiae*, known as brewer’s or baker’s yeast, is common in the environment on tree bark and rotting wood as well as a commensal in the human gastrointestinal tract (Wang *et al.* 2012; Murphy and Kavanagh 1999), leading to frequent opportunities to function as a pathogen. Indeed, *S. cerevisiae* has been shown to occasionally cause invasive, disseminated infections in immuno-compromised individuals as well as vaginitis in nonpredisposed individuals (Enache-Angoulvant and Hennequin 2005; Murphy and Kavanagh 1999). Surprisingly, despite its utility as a genetic model, *S. cerevisiae* has been underutilized in empirical studies on evolution of virulence and relatively little is known about the genetics underlying its pathogenicity (Goldstein and McCusker 2001). Clinical isolates of *S. cerevisiae* are reported to show certain phenotypes including the ability to grow invasively at high temperatures (>39°), copper resistance and pseudohyphal growth, suggesting that specific alleles underlying these phenotypes contribute to fitness of *S. cerevisiae* *in vivo* (McCusker *et al.* 1994; Strope *et al.* 2015). Other studies, however, have not demonstrated a clear division of clinical isolates of *S. cerevisiae* either genetically or phenotypically (Klingberg *et al.* 2008), suggesting that the genetics behind opportunistic pathogenicity in *S. cerevisiae* is complex.

Here, we combine the genetic tools of *S. cerevisiae* with an invertebrate infection model to understand variation in pathogenicity within the species and to identify its underlying genetic basis. We screened a diverse pool of genotypes of *S. cerevisiae* for their ability to survive and grow inside the larvae of the wax moth, *Galleria mellonella*, which is a model host often used in studies of microbial pathogenesis (Kavanagh and Reeves 2004; Mylonakis *et al.* 2005). Our goals were to identify genotypes that outcompeted others *in vivo* and to test whether clinical isolates generally have high pathogen potential. Because *S. cerevisiae* has not been documented to be naturally associated with *G. mellonella*, our choice of host represents a novel and challenging environment in which the fitness of the genotypes does not reflect historical adaptation to the host but instead selects for pathogenicity factors of a generalist nature. We also performed bulk segregant analysis (BSA) (Parts *et al.* 2011) to determine the number of quantitative trait loci influencing pathogenicity. Lastly, we conducted an additional passaging study using the library of mutants in the *S. cerevisiae* deletion collection. This approach allowed us to test the contribution of most genes in the *S. cerevisiae* genome to pathogenicity separately in a controlled genetic background and to identify types of genes that are required during infection of the insect host. By leveraging natural and engineered variation in a model fungus, we were able to describe the genetic architecture of pathogenicity toward a model host that draws deep parallels to other opportunistic fungi.

## Materials and Methods

### Strains

Strains used in virulence and quantitative trait locus (QTL) mapping experiments are shown in Supplemental Material, Table S1. The 43 diploid strains derived from wild-type isolates used for serial passaging experiment I are shown in Table S2. Each of these strains was constructed using methods described previously (Maclean *et al.* 2017). Briefly, wild isolates (Liti *et al.* 2009; Schacherer *et al.* 2009) were barcoded by replacing the *HO* locus with a cassette containing two unique 20 bp barcodes (uptags and downtags) and a g418 resistance marker allele *KANMX4* (Table S2). These heterozygous diploids were sporulated on potassium acetate media, and *MATa* and *MATα* G418 ho::*KANMX4* colonies were obtained and mating type determined using a PCR assay (Huxley *et al.* 1990) with three primers: OSP37 (5′-TGGGCAGTTTACCTTTACGG), OSP38 (5′-TGTCTTCTCTGCTCGCTGAA), and OSP39 (5′-GCAAAGCCTTAATTCCAAGG). The drug resistant marker of *MATa* strains was switched to hygromycin B, and *MATa* ho::*HYG* and *MATα* ho::*KANMX4* strains were mated to form diploids. Experimental passaging experiment II utilized a second subset of strains from the 4828 haploid strains of *MATα* from the *S. cerevisiae* deletion collection (Winzeler *et al.* 1999). Each strain in this set has a unique open reading frame replaced with a cassette containing two distinct 20 bp barcodes flanking a g418 resistance marker allele (Giaever *et al.* 2002).

### G. mellonella infection protocol

*G. mellonella*, the greater waxmoth, is a model host for studying fungal pathogenesis (Fuchs *et al.* 2010). We maintained a healthy growing colony of the host maintained at 30° on a diet containing ∼400 g of Gerber multigrain baby formula, 100 ml corn syrup, 100 ml glycerine, 50 ml tap water, and 1–2 ml of the antimicrobial propionic acid. In cases when the colony did not provide enough hosts, we used waxworms obtained from the first available supplier of fishing baits. Inoculations were performed by injection into the final posterior left proleg of last instar larvae using cells in 10 μl H_2_O, after washing by pelleting cells, resuspending in H_2_O, and repelleting. Sham inoculations using H_2_O were used to control for background variability in larval response to trauma, excluding results when control animals died. All inoculated larvae were stored at room temperature (20–22°) under ambient lighting.

### In vivo generation time estimates

We used a haploid strain (SSP253) derived from the wild diploid clinical isolate 322134S (Table S1) to estimate the generation time of *S. cerevisiae* *in vivo*. We grew the frozen stock of the strain on YPD (Yeast extract Peptone Dextrose) agar containing g418 (200 μg/ml) and transferred cells to 3 ml of liquid YPD medium for overnight growth at 30°, with shaking at 200 rpm. The next day, we washed the overnight culture, adjusted the density to 10^9^/ml with sterile water, and used it to inject 25 larvae, each with 10 μl. We crushed three larvae immediately in 30 ml sterile water after injection and subsequently every 4 hr for 48 hr to recover yeast cells. At every time point, the crushed extract was serially diluted and plated on YPD agar containing ampicillin (100 μg/ml), chloramphenicol (17 μg/ml), and g418 (200 μg/ml). We counted the colony forming units after 48 hr of incubation at 30° to estimate the density recovered from the host at each time point. These data were used to plot a growth curve to estimate the generation time assuming exponential growth after the initial postinjection bottleneck (Figure S1; time points 16, 24, 32, and 40 hr).

### Serial passaging experiment I

We grew a consortium of 43 wild strains in two separate conditions: in YPD medium (*in vitro*) and injection into waxmoth larvae (*in vivo*).

#### Inoculum preparation:

Inoculum was prepared after reviving the strains from frozen stocks by inoculating YPD agar containing g418 (200 μg/ml) followed by overnight growth at 30°. The colonies were used to initiate cultures in 3 ml liquid YPD media that were grown overnight at 30° with shaking. The density of individual strains was measured using a hemocytometer and adjusted to 10^8^ cells/ml prior to mixing them in equal volumes and adjusting to the final density of 10^9^/ml using sterile water. Eight independent mixtures were made for each replicate founding population each of which was used to found both an *in vitro* and *in vivo* population. Frozen stocks of the initial inocula were made in 20% glycerol and stored at −80°.

#### In vivo passaging:

We initiated eight replicate populations of five larvae. Each larva was injected with 10^7^ cells. Five larvae were injected with sterile water as a control. After 48 hr, yeast cells in each population were collected by crushing the five larvae together in a mortar and pestle and resuspending the extract in 50 ml sterile water, followed by filtration through sterile nylon (40 μm) to separate the yeast cells from the larval tissue. After filtration, 2 ml of the extract was mixed with 8 ml liquid YPD medium containing g418 (200 μg/ml), ampicillin (100 μg/ml), and chloramphenicol (17 μg/ml), and grown overnight at 30° with shaking at 200 rpm. The next day, the cultures were pelleted and washed with sterile water and their density adjusted to 10^9^/ml using hemocytometer counts prior to injecting for the next passage. We carried out a total of 10 passages. We also estimated the density of yeasts recovered from each replicate population after each *in vivo* passage. For this, we diluted the filtered extract serially with sterile water and plated on YPD agar containing drugs at above concentrations. The density of recovered cells per larva was estimated using the number of colony forming units obtained after 48 hr of incubation at 30°.

#### In vitro passaging:

We used YPD liquid media containing the drugs at the concentration mentioned above for serial passages *in vitro*. Each of the eight replicate populations was initiated by transferring 200 μl of the inoculum at the density of 10^9^/ml to a total of 1 ml medium. Each subsequent passage was initiated by transferring 200 μl culture from the previous passage after adjusting the density to 10^9^/ml to a total of 1 ml fresh medium. The populations were grown overnight at 30° with shaking. The *in vitro* passaging was also conducted for a total of 10 transfers or ∼40 generations.

### Serial passaging experiment II

We grew a subset of the *MATα* strains of the *S. cerevisiae* deletion collection in both *in vivo* and *in vitro* conditions as described for passaging experiment I. Procedures for inoculum preparation and passaging were identical unless listed below.

#### Inoculum preparation:

Colonies obtained from growing the frozen stocks were used to initiate cultures in 1 ml liquid YPD media in 96-deep-well plates and incubated overnight at 30° with shaking at 200 rpm. Densities of strains in the deletion set were not equalized, but instead were mixed using equal volumes and adjusting to the final density of 10^9^/ml using sterile water.

#### In vivo passaging:

After filtration of larval extracts, 20 ml of the extract was mixed with 30 ml liquid YPD containing antibiotics and grown overnight at 30° with shaking at 200 rpm. We carried out a total of three passages.

#### In vitro passaging:

The populations were initiated by transferring 2 ml of the initial populations to a total of 5 ml medium. The populations were then transferred for 10 passages at the same rate, to reflect the *in vivo* passaging portion of the experiment.

### Illumina sequencing of barcodes

We used a Qiagen Pure Yeast Gene kit to extract DNA for sequencing the 20 bp unique barcodes. For passaging experiment I, DNA was extracted from the initial inocula and from replicate populations after the first, fifth, and 10th *in vitro* and *in vivo* passages. For passaging experiment II, DNA was extracted from the first, fifth, and 10th passages *in vitro* and the first, second, and third passages *in vivo* in addition to the initial inocula. To obtain enough high-quality DNA for the passages *in vivo*, we used the cultures obtained after overnight growth of the filtrates collected after the respective passages.

We prepared one Illumina library for each replicate population at each passage in each environment using a barseq (barcode sequencing) approach similar to Smith *et al.* (2010), by amplifying the uptag barcodes to obtain ∼50 ng/μl of 186 bp PCR product using Ex Taq DNA polymerase (Takara). The primers fused the Illumina adapters to the amplification primers flanking the uptags, OSP12: 5′-AATGATACGGCGACCACCGAGATCTACACTCTTTCCCTACACGACGCTCTTCCGATCTNNNNNNGATGTCCACGAGGTCTCT, and BSeqR: 5′-CAAGCAGAAGACGGCATACGAGATXXXXXXGTGACTGGAGTTCAGACGTGTGCTCTTCCGATCTGTCGACCTGCAGCGTACG, where *N* is a mixture of all bases and X is a unique 6 bp sequence used for multiplexing. The products were purified using a Qiagen PCR purification kit before sequencing using a multiplexing approach through Illumina Hiseq-2000 50 bp single-end platform at the University of Michigan DNA Sequencing Core. The libraries, *i.e.*, replicate populations, from the two experiments were divided across three Illumina lanes. Each library was sequenced to an average total depth of 5.08 × 10^6^ reads (range, 1.62–9.72 × 10^6^). On average, we obtained ∼4,200,000 reads per library that were assignable to genotypes for both passaging experiments, with a mean of 9.5 × 10^4^ reads per genotype for experiment I and 1.0 × 10^3^ reads per genotype for experiment II.

### Analysis of barseq data from serial passaging experiment I

We used a previously described script (Heisler 2012) to calculate frequencies of strain-specific barcodes from reads obtained through Illumina sequencing to estimate relative fitness of the strains *in vivo* and *in vitro*. Specifically, for each library corresponding to a replicate population at a given time point and environment, we trimmed each 50 bp Illumina single-end read by removing the first 24 and last eight nucleotides and retained the remaining 20 bp corresponding to barcode sequences. This 20 bp region was aligned with barcodes in the strain-specific barcode map (Table S2) allowing a maximum of two mismatches to account for the inherent errors during DNA sequencing. Alignments with a score above 90 (on a scale of 0–100) were used to assign the corresponding sequence read to a unique strain. We discovered that one strain was inadvertently outcrossed instead of selfed as planned (SSP81). This strain appeared to be a mating product of two strains: 322134S, a clinical strain from Newcastle, UK and YPS606, an oak bark strain from Pennsylvania. In order to separate the barcodes from SSP81 from YPS606, we confirmed that by the end of the serial passaging experiment, all of the clones were doubly barcoded by colony PCR of >190 clones. Using the ratio of the two barcodes, we estimated that each barcode (YPS06) = 1.75 × barcode (322134S). Correcting for this allowed us to approximately separate SSP81 from YPS606 in both *in vivo* and *in vitro* passages. Where estimated sequences of YPS606 were negative due to the correction, a value of zero was used. On average, we were able to assign ∼82% reads per library. The total number of reads obtained for a strain in a given library was used in calculation of frequencies for strains that had >100 reads assigned to them in the initial inoculum.

The fitness of the strains in specific environments was approximated using fold-change (*F*) between initial and after the first passage using the formula:

$$F=\frac{S_{n}T_{n-1}}{S_{n-1}T_{n}}-1,$$ (1)

where *S_n_* and *S_n_*_−1_, are the total number of reads assigned to strain *S* at the *n*th and previous passage, respectively, and *T_n_* and *T_n_*_−1_ are the total number of reads across all strains in the corresponding libraries. The effect of isolation source, *e.g.*, habitat, on *F* was tested using ANOVA for both environments.

### Analysis of barseq data from serial passaging experiment II

The libraries from the deletion collection were demultiplexed using barcodes downloaded from the *Saccharomyces* Genome Database. Barcodes found <100 times in any of the initial eight populations were eliminated, leaving a total of 4110 genotypes for analysis. We calculated *F* for each mutant as above, and used these values to test for correlation between time points using Pearson’s *r*. In order to find mutant genotypes whose relative fitness differed between *in vitro* and *in vivo* conditions, we tested for differential abundance between the sets of replicate populations using an exact test with a negative binomial model with dispersion as implemented in edgeR (Robinson *et al.* 2010). We adjusted *P*-values using the Benjamini–Hochberg method to control the false discovery rate at 5%. The large number of mutant genotypes of significantly different fitness were characterized functionally for differences in gene ontologies using the standard hypergeometric test of the GOStats package (Falcon and Gentleman 2007). The universe of possible terms was derived from the 4110 genotypes we successfully recovered from the initial populations, and the exploratory gene sets were those that were either significantly more fit in the *in vivo* or in the *in vitro* passages. The output of GOStats was plotted with the aid of the GOplot package (Walter *et al.* 2015).

### Pseudohyphal filamentation assays

Strains were grown overnight in 5 ml liquid YPD. For assays of surface filamentation, a dilution of the overnight culture containing roughly 100 cells was spread on synthetic low ammonium dextrose agar medium (0.17% yeast nitrogen base without amino acids or ammonium sulfate, 2% dextrose, and 50 μM ammonium sulfate). Plates were incubated for 5 d at 30° and single colonies were imaged. Pseudohyphal filaments are evident circling the colony periphery. Conditions of low nitrogen were used to induce surface-spread filamentation, as similar conditions have been found to induce pseudohyphal growth efficiently in the Σ1278b strain of *S. cerevisiae*.

### Virulence estimation

We measured relative pathogenicity of individual strains for two features. Virulence was estimated using host mortality caused by individual strains following injection, and the relative ability to survive and proliferate in the host was measured using the relative proportion of the injected yeast recovered after 48 hr from inside the host in pairwise competition with another strain.

#### Host mortality:

Frozen stocks of the haploid strains derived from the wild isolates YPS606, 322134S, and YPS128 (Table S1) were grown separately at 30° on YPD agar plates containing g418 (200 μg/ml). For each strain, inoculum was prepared by overnight growth with shaking at 30° in 3 ml liquid YPD containing g418 at the same concentration. The cells were washed and the density was adjusted to 10^9^/ml using sterile water after hemocytometry. This inoculum was used to inject 10 larvae per strain with 10 μl per larva. The number of dead larvae was recorded for 7 d to estimate percent mortality by each strain. A population of 10 larvae, each injected with sterile water was used as a control.

#### Yeast recovery following two strain coinjection:

We used haploid strains of YPS606, 322134S, and YPS128 and L-1528 genetic background to estimate virulence by pairwise competition (Table S1). The strains were sporulated for 3 d in minimal media at room temperature to isolate haploid progeny of *MATα*. The ploidy of the progeny was verified using the PCR approach as described above. Each of these haploids contained a g418 resistance marker allele at the *HO* locus, which was replaced with an allele conferring resistance to nourseothricin (*NAT*) to create nearly isogenic pairs of wild strains that differ only by the drug marker (Table S1).

The eight haploid strains (four wild genetic backgrounds × two drug markers) were grown overnight by shaking at 30° in 5 ml liquid YPD containing g418 (200 μg/ml) or NAT (100 μg/ml). The cultures were washed with sterile water the next day and the densities were adjusted to 10^9^/ml. Equal volumes of strains were mixed with each other to create pairwise combinations in which the two strains in a pair represented a distinct genetic background of their wild parental isolate and a distinct drug resistance marker. The initial density of each strain in the mixture was obtained by plating the pairwise mixture separately on YPD agar containing ampicillin (100 μg/ml) and chloramphenicol (17 μg/ml) along with either g418 (200 μg/ml) or NAT (100 μg/ml), prior to injection. The number of colony forming units 48 hr after incubation at 30° were used to estimate initial density per milliliter.

Three larvae (10 μl/larvae) were injected per pairwise combination per replicate and data were obtained from a total of four replicates. For each replicate, the yeast cells were recovered by crushing the three larvae into 30 ml sterile water 48 hr after injection. The extract was serially diluted and plated on the same agar media as described above to obtain colony forming units for each strain in the pair based on the drug marker. The number of colony forming units was counted after 48 hr of incubation at 30° to estimate relative recovery of the strains per ml and per larva. For each pair, differences between the final and initial relative density of each strain out of total were compared with the initial relative density of the strain in the given replicate.

### BSA

To investigate the genetic basis of differences in virulence and survival *in vivo* between 322134S and YPS128, we performed an analysis of quantitative trait loci using the BSA approach. A clone of *MATa* in the YPS128 background (SSP96) was isolated and tagged with a cassette containing a hygromycin resistance allele and an RFP marker linked to the *MATa* allele at the mating type locus (Chin *et al.* 2012). The derived strain (SSP264) was crossed with a clone of *MATα* isolated from the background of 322134S (SSP253). The resulting diploids were identified using PCR as detailed above. The diploids were sporulated as explained above and spores were separated using zymolyase followed by sonication (Rockmill *et al.* 1991). An Aria II flow cytometer was used to separate the RFP-labeled spores of *MATa* from the unlabeled *MATα* spores to obtain a nearly pure haploid population in which all progeny were *MATa* and unable to mate among each other. Approximately 10^5^ RFP-labeled spores from the sorting were plated on YPD agar plates containing hygromycin (200 μg/ml) at 30° for 48 hr, after which they were collected into sterile water at the density of 10^9^ cells/ml. This initial inoculum was used to initiate serial passages *in vivo* and *in vitro*.

For *in vivo* passages, 10 μl of the inoculum from each cross was injected into each of five larvae and three serial passages *in vivo* were conducted using a single replicate and a similar strategy as explained above (see the section *Serial passaging experiment I*). Briefly, the host larvae were crushed in 50 ml sterile water 48 hr after injection, and the extract was filtered using a sterile 40 μm nylon filter. Yeast cells from 2 ml of the filtrate was combined with 8 ml of YPD containing hygromycin (200 μg/ml) and antibacterial drugs at the same concentration as previously mentioned, and grown overnight with shaking at 200 rpm at 30°. The next day, the cells were washed, adjusted to 10^9^ cells/ml, and 10 μl was injected into five new larvae for the next passage. The overnight growth in YPD after the third passage in the host was washed with sterile water and used to extract DNA using a Pure Gene Yeast kit. Genomic DNA libraries were prepared by fragmenting DNA using a Covaris ultrasonicator and a TruSeq (Illumina) library kit, and sequenced on one lane of an Illumina HiSeq-2000 platform to obtain 1.28 × 10^8^ 50 bp single-end reads. Sequencing of haploid parents of the BSA cross between 322134S and YPS128 was performed using the Nextera XT kit (Illumina) with sequencing on a portion of a HiSeq-4000 lane with paired end 150 bp reads yielding 7.6 × 10^6^ average read pairs per strain.

We also carried out three serial passages *in vitro*. Specifically, 1 ml of initial inoculum of germinated spores from each cross was added to 9 ml liquid YPD containing hygromycin (200 μg /ml) and incubated overnight at 30° with shaking at 200 rpm. Subsequent passages involved addition of 9 ml fresh medium to 1 ml of the overnight grown populations every 24 hr for 3 d. Genomic DNA libraries were prepared as described above for populations obtained after the third passage *in vitro* as well as the initial inocula.

### QTL analysis

We combined the BSA initial, *in vitro*, and *in vivo* passages with raw sequence reads of SSP96 and SSP265 to identify segregating SNPs. The data were trimmed using trimmomatic 0.33 with settings (HEADCROP:6 LEADING:33 TRAILING:27 SLIDINGWINDOW:4:28 MINLEN:30) (Bolger *et al.* 2014). Reads were aligned to S288c with bwa-mem (Li 2013). We then used the standard workflow for SNP identification using GATK v 3.6 (McKenna *et al.* 2010). We used the vcf file of Bergström *et al.* (2014) to recalibrate bases using GATK’s BaseRecalibrator. After SNP calling yielded 88,306 SNPs, we used HaplotypeCaller and then filtered the SNPs with VariantFiltration with the following parameters: –filterExpression “QD < 5 || FS > 50.0 || MQ < 50.0 || MQRankSum < −12.5 || ReadPosRankSum < −8.0.” This reduced the data set to 80,593 SNPs. The parental haploid genomes were assembled after trimming using trimmomatic with commands as above (except HEADCROP:4 TRAILING:26 MINLEN:50) using velvet 1.2.10 (Zerbino and Birney 2008), with hash length optimized using VelvetOptimizer.pl. Because we had the initial population and the parental genomes, we were able to further filter the data to only include those in which the two parents were predicted to be homozygous for different genotypes and for which the initial population was polymorphic, but with an allele frequency of major allele frequency ≤0.85. We also filtered SNPs of Phred-scaled quality <5000 or if any of the BSA pools (initial, *in vitro*, or *in vivo*) had a depth of <30 reads. This yielded 44,153 SNPs for analysis.

Estimates of allele frequency of each pool and logarithm of the odds (LOD) scores for QTL between *in vivo* and *in vitro* pools were estimated using MULTI POOL (Edwards and Gifford 2012) using contrast mode. We used *n* = 1000 (number of individuals per pool), assuming a 99% bottleneck of the population during passaging, although this number likely differs between *in vitro* and *in vivo* conditions. MULTI POOL allows the 90% confidence window for each QTL (here considered a LOD score >5) to be estimated as well as the most likely QTL position, using a Bayesian framework that simultaneously incorporates all linked SNPs. Allele frequencies for each pool were estimated by running each pool against itself in contrast mode. BSA observed and estimated SNP frequencies were plotted using the ggplot2 package of R (Wickham 2009).

### Data availability

Strains are available upon request. Code for figures and data files have been archived at https://github.com/Michigan-Mycology/Saccharomyces-barseq. Raw sequence data has been deposited into NCBI’s SRA archive under BioProject: PRJNA390197, with the accession numbers SRR5684247–SRR5684364.

## Results

### Saccharomyces cerevisiae kills G. mellonella and multiplies in vivo

**Figure 1:**
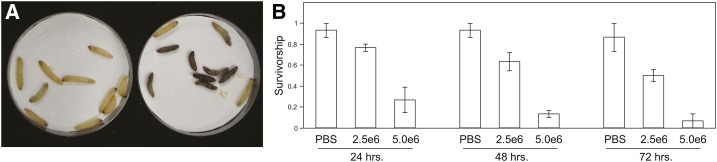
*S. cerevisiae* causes significant mortality to *G. mellonella* larvae. (A) At 4 hr after injection of 5 × 10^6^ cells of BY4722 in 10 μl PBS into 10 *Galleria* sixth instar larvae, animals show evidence of melanization and morbidity (right dish), whereas controls injected with 10 μl PBS appear healthy. (B) Survivorship for 72 hr is significantly different (*P* < 0.05) among treatments (PBS control, 2.5 × 10^6^ cells per animal, and 5 × 10^6^, Cox proportional hazards test)_,_ Error bars indicate SD across three replicate populations of 10 larvae per treatment.

We observed that larvae injected with a fatal dose of *S. cerevisiae* turned a black color (consistent with melanization) within 30 min postinjection as opposed to worms injected with PBS or H_2_O (Figure 1A). The survivorship of the larvae postinjection was observed to be dosage-dependent (Figure 1B). Despite the consistent negative effects of the *S. cerevisiae* inoculation on the larvae, we observed that a large proportion (∼99%) of the yeast cells were killed within 1 hr by the host, based on recovery of cells immediately determined postinjection. However, the cells were able to divide within the host following the bottleneck as evidenced by the estimated doubling time of 8 hr (Figure S1). Although the negative effects on larvae were consistent, in general the estimates of relative virulence of strains using host survivorship measured on pools of larvae inoculated with a single strain were difficult to repeat. To overcome these challenges, we used coinfection experiments that eliminate many of the variables, presumably due to intrahost individual and environmental factors that we are unable to control. In order to maintain a large population size after infection to facilitate passaging and minimize genetic drift, larvae were inoculated with an overdose (10^7^ cells per larva), deceased larvae crushed after 48 hr, the yeast multiplied overnight by addition of YPD medium plus antibiotics to the extract, and finally, the yeast cells were harvested, washed, and adjusted to the appropriate density for the next round of injection. Over passages, we observed an increased accumulation of melanin in the larval extract which was negatively correlated with recovery of cells.

### Fitness of Saccharomyces cerevisiae genotypes differs in vivo and in vitro

Serial passaging experiment I used a consortium of wild *S. cerevisiae* genotypes passaged through two distinct environments: *in vivo*, using a model host, *G. mellonella*; and *in vitro*, using a standard yeast growth medium, YPD. We estimated fitness of genotypes in both environments using their relative frequency, which was obtained from sequencing genotype-specific barcodes from our initial populations as well as after specific passages. Of 43 barcoded genotypes used in passaging experiment I, 10 represent those derived from clinical isolates, whereas the rest represent isolates from various *S. cerevisiae* habitats and one represents a laboratory hybrid (which was inadvertently included) between a clinical and oak bark strain (Liti *et al.* 2009; Maclean *et al.* 2017). We hypothesized that one or more clinical isolates will likely have higher fitness in the novel host as compared with nonclinical genotypes, if our model host provides some of the same selective pressures that the pathogen can experience in a mammalian host. To test these hypotheses, we analyzed how relative frequencies of the genotypes changed in replicate populations after the first, fifth, and 10th passage in each environment.

**Figure 2:**
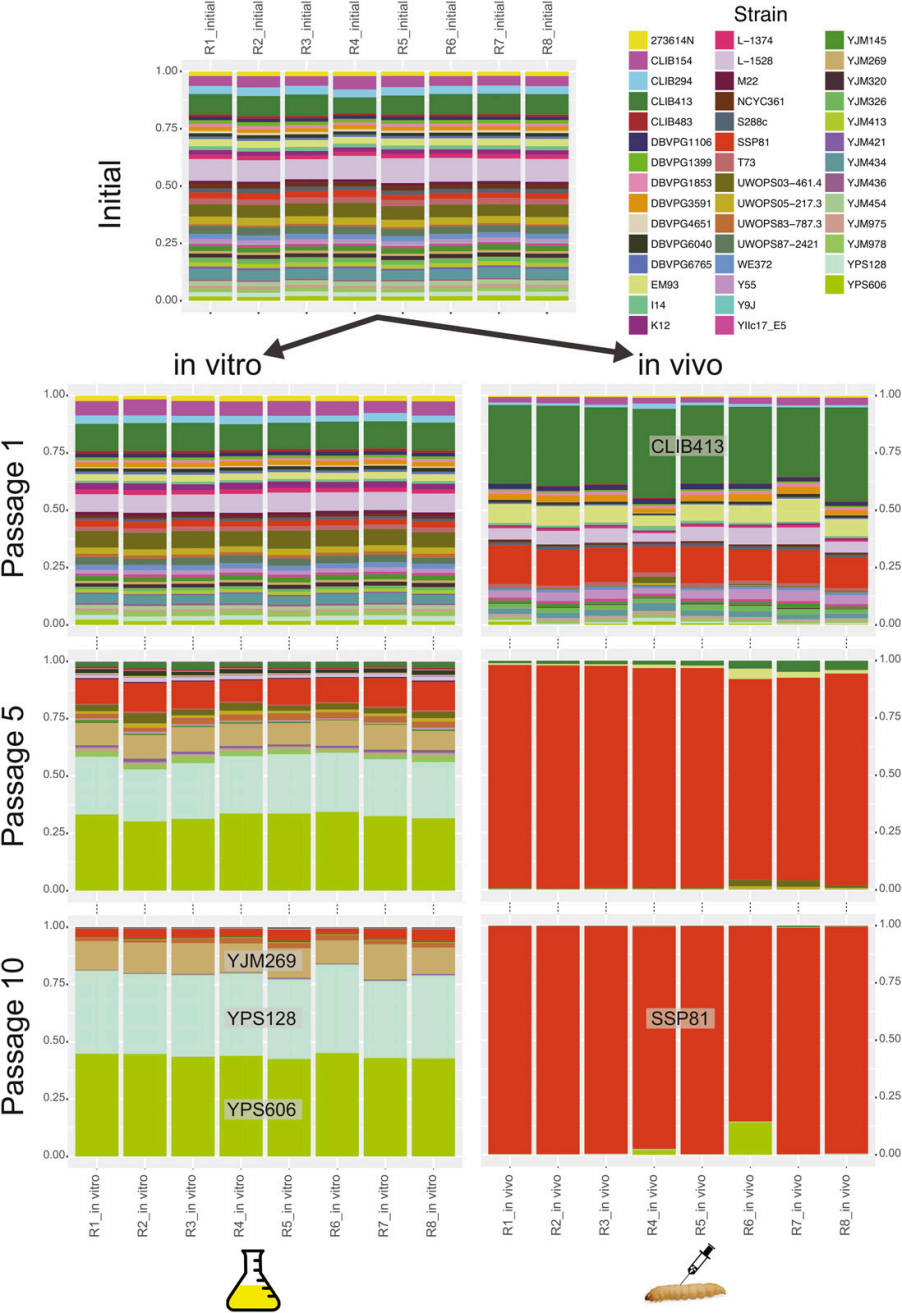
Change in relative frequencies of the 43 genotypes in serial passaging experiment I. Each stacked bar plot represents the proportion of the barcode sequences recovered that could be assigned to one of the strains. In the top panel, the frequencies of the eight initial populations are shown. These samples were used to start both *in vitro* and *in vivo* passages.

During the course of 10 passages *in vitro* during experiment I, the frequencies of the 43 genotypes changed dramatically and consistently across replicate populations (Figure 2). After a single passage, the initial diversity was not greatly changed, but some strains, such as CLIB413, noticeably increased in frequency. However, by passage 5, three strains emerged as dominant (YJM269, YPS128, and YPS606), and by passage 10 these same strains were even more dominant, comprising >89% of the populations. The *in vivo* passaging of experiment I showed a more rapid loss of genotype diversity (Figure 2). After a single passage, strain CLIB413 showed a dramatic increase (mean 35% across populations), but by passage 5, this genotype was reduced to a low frequency (mean 3%). At passage 5, the hybrid strain (SSP81) represented most (>94%) of the barcodes recovered, and by passage 10, strain SSP81 represented nearly all of the reads excepting some uncertainty due to mathematical error correction for the duplicated barcode in the hybrid and pure strain. The hybrid strain SSP81 is a cross between 322134S, a clinical isolate from Newcastle, UK, and YPS606, an oak bark isolate from the USA, and was inadvertently included in our pool. Below we perform additional experiments to determine the genetic contributions leading to the differential success of SSP81 in the larval habitat.

**Figure 3:**
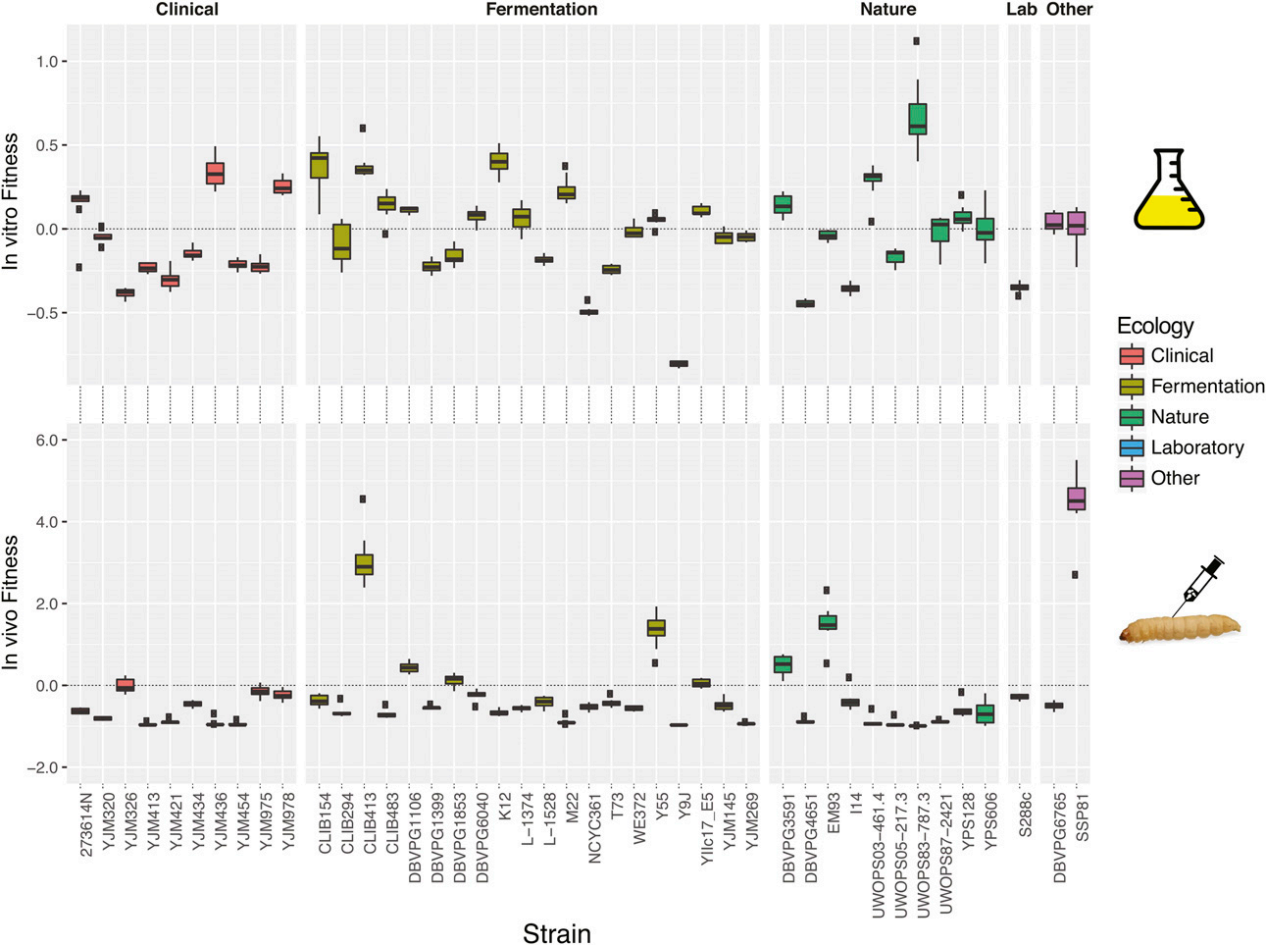
Success in *in vivo* and *in vitro* habitats in passaging experiment I is not correlated with source of isolation. In the top and bottom panels are shown the fitness of the 43 strains measured after a single passage grouped by isolation source in the *in vitro* and *in vivo* environments, respectively. Fitness is measured as the fold change from the initial to after the first passage. Boxes indicate 25th–75th percentiles (IQR), and whiskers extend to the points within 1.5× the IQR. Outlier points are shown as separate dots.

In order to test whether clinical isolates performed better than isolates from other habitats, we used fitness as assessed after the first passage *in vivo* and *in vitro* (Figure 3). There was no correlation of relative fitness of genotypes across the two environments (*r* = 0.15, *P* = 0.33). The source of isolation was not a significant factor (one-way ANOVA excluding SSP81, S288c, and DBVPG6765) determining fitness in either *in vitro* ([*F*(2,37) = 0.30, *P* = 0.75]) or *in vivo* environments ([*F*(2,37) = 0.91, *P* = 0.41]).

**Figure 4:**
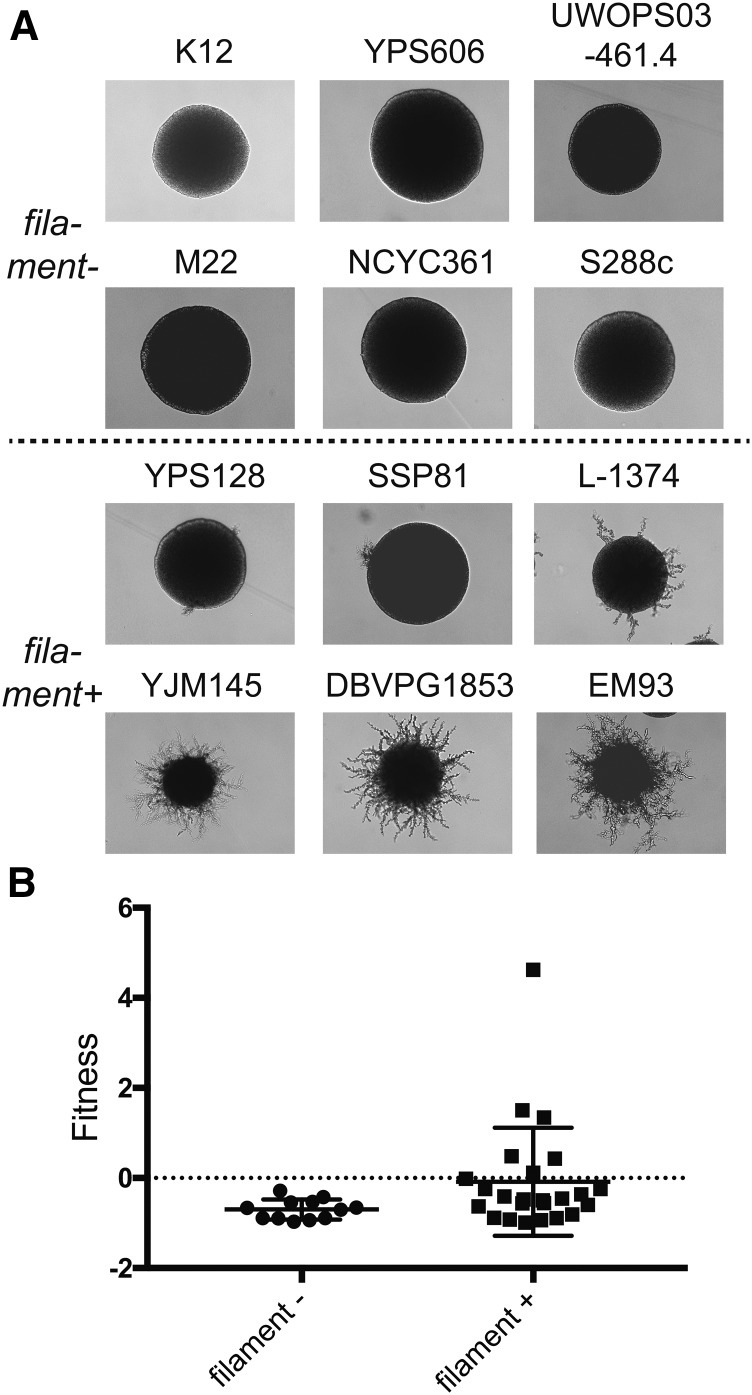
The ability of strains to grow filamentously influences fitness in *G. mellonella*. (A) Representative photos of strains without or with evidence of filamentous growth on SLAD media after 5 d and photographed at low magnification. (B) Comparison of fitness measured as fold-change (*F*) in *G. mellonella* with the ability to grow filamentously. Horizontal lines show means and whiskers show SD. Differences between means were significant (*P* = 0.03) tested using Mann–Whitney *U*-test (used due to unequal variance between classes).

Pseudohyphal growth is strongly linked to pathogenicity and virulence in several fungi, including the opportunistic human fungal pathogens *C. albicans* and *Cryptococcus neoformans* (Wang *et al.* 2002; Leberer *et al.* 1996). Thus, pseudohyphal formation may also be involved in persistence or host evasion in the case of *G. mellonella*. We therefore tested the ability of our wild strains to undergo pseudohyphal growth according to standard methods (Gimeno *et al.* 1992). As indicated in Figure 4A, the strains were cultured under conditions of nitrogen limitation to induce surface-spread filamentation. We then correlated the ability of these strains to form pseudohyphal filaments with *in vivo* fitness measured in serial passaging experiment I. These results showed that filamentation (*P* = 0.03, Mann–Whitney *U*-test) in our assays was positively associated with pathogenicity in *G. mellonella* (Figure 4B).

### Genetic analysis of in vivo fitness in the hybrid strain

Passaging experiment I identified strain SSP81 as being specifically fit in the *in vivo* habitat. To understand the genetic components of pathogenicity in this hybrid isolate, we evaluated pathogenicity of the two haploid parental components (322134S and YPS606) using pairwise interactions by coinjection, followed by estimating recovery of the two genotypes using selective media. We also included comparisons with one strain YPS128, which was only found to perform well *in vitro*, and strain L-1528, which performed poorly *in vivo* and *in vitro*. The results confirmed the relative fitness of the genotypes *in vivo*, with strain 322134S outperforming the other three genotypes, and significantly so against L-1528 and YPS128 (*t*(6) = 5.7, *P* = 0.001 and *t*(6) = 4.2, *P* = 0.006, respectively) (Figure S2). In three of the four replicate coinjections with 322134S and YPS606, strain 322134S outcompeted YPS606, but the fitness was not significantly different over the four replicates. Strain YPS606 also did not perform better than YPS128 and L-1528 *in vivo*, suggesting overall that the 322134S genotype has higher pathogenicity than YPS606. To support these data, we performed virulence experiments estimating the survivorship of larvae postinoculation with single strains. Despite the high variation among replicate populations in this assay, there were significant differences among strains, with clinical isolate 322134S consistently causing significantly higher host mortality (one-way ANOVA, *F*(2,8) = 9.2, *P* = 0.02), killing an average 90% of the hosts in a week compared with the YPS606, which caused an average of only 50% mortality (Figure S3). There was no significant difference between the host mortality caused by strains 322134S and YPS128 or between YPS606 and YPS128 (Tukey’s HSD).

We then used BSA to identify QTL potentially related to *in vivo* fitness differences among these strains. Haploid strains 322134S and YPS128 were crossed, the diploid strain (SSP267) sporulated, and ∼10^5^ meiotic progeny isolated and passaged independently both *in vitr*o and *in vivo*. We insured that only haploid cells were present by tagging the *MATa* locus with a red fluorescent protein and isolation of red fluorescing spores by flow cytometry. After passaging, the two passaged populations and the initial population before passaging were subjected to whole genome sequencing to reveal QTL indicating differential selection in the two conditions. We considered only SNPs that were predicted to be polymorphic based on the genome sequences of the parents and that had a major allele frequency <0.85 in the initial population. This resulted in 44,153 validated SNPs segregating at an average depth of 132X for the initial population, 135X for the *in vitro* passaged, and 83X for the *in vivo* passaged.

**Figure 5:**
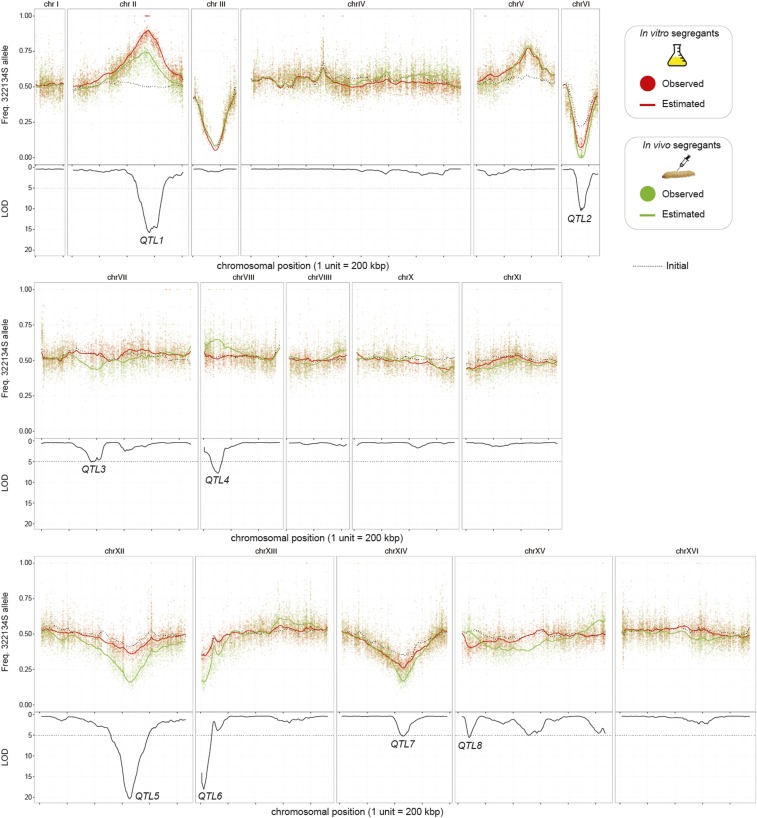
Bulk segregant analysis identifies eight QTL regions differentially affecting survivorship *in vitro* *vs.* *in vivo*. Each panel indicates a different chromosome with the top portion the frequency of the allele from the 322134S parent and the lower portion the LOD score. Black dashed line indicates the estimated allele frequency of the initial pool before passaging. Green dots indicate estimated SNP after three passages *in vivo*, and the green line indicates the estimated frequency of the bulk population. The red dots and lines indicate the corresponding values after three *in vitro* passages. Eight regions surpassing LOD 5.0 are labeled as QTL and discussed in the text.

The allele frequencies in the bulk selected by *in vivo* passaging changed more than the *in vitro* only passaging. The mean frequency of the alleles from the 322134S parent was 0.510 initially. This changed to a mean of 0.513 in the YPD selected pool *vs.* a mean of 0.497 in the larvae selected pool. Overall, the size of the genomic regions differing from equal segregation, *i.e.*, 0.5, were large, occasionally as big as some of the smaller chromosomes, suggesting that our population sizes were considerably lower than the number of spores with which we initiated our populations (Figure 5). Chromosome III, which contains the mating type locus, showed very biased segregation toward the YPS128 parent, showing that the isolation of *MATa* spores by flow cytometry was successful. Other genomic regions, such as chromosome V, also showed biased segregation of both populations from 0.5. As opposed to the mating type chromosome, this selection was imposed after the passaging as evidenced by deviation from the initial population frequencies.

Eight regions showed allele frequency differences between *in vitro* and *in vivo* populations with a LOD score >5.0. The QTL fell into two main groups. In the first group both *in vitro* and *in vivo* populations differed from the initial in the same direction (QTL1, QTL2, QTL5, QTL7; Figure 5). This pattern supports a favored allele that is beneficial in both environments. The second group are those in which only one population differed from the initial (QTL3, QTL4, QTL6, QTL8). The expectation was that the favored allele in the *in vivo* population would be derived from the 322134S parent, but this was only the case for QTL4 and QTL8. The strongest QTL effect sizes were ones in which the alleles from the YPS128 parent were favored (QTL1, QTL5, QTL6).

The MULTI POOL method estimates the QTL region with the maximum likelihood (sublocalized best location), but also produces confidence intervals for each QTL. The 90% credible intervals for the eight QTL ranged from 24.4 kp (QTL6) to 969.1 kb (QTL8). The 90% credible intervals comprising QTL contained a minimum of 12 genes to over 100 genes. The genes closest to each of the QTL best locations were *ICS2*, *SEC4*, *DBP3*, *STE20*, *YEF3*, *COX14*, YNL095C, and *NOP8*, respectively for QTL 1-8. QTL4 is of particular interest as it is the only QTL which shows an increase of the *in vivo* population for the 322134S allele above the *in vitro* and initial (Figure 5), and the gene nearest the QTL’s best location, *STE20*, is widely implicated in virulence in other opportunistic fungi (Calcagno *et al.* 2004; Wang *et al.* 2002). However, the QTL4 50% credible region contains seven genes in a span of 21.8 kb. This region contains 132 SNPs and seven gapped regions differing between 322134S and YPS128. All of the genes had SNPs, and most genes had amino acid replacing polymorphisms, making it impossible to determine the alleles underlying this QTL with the current data set.

### Identification of genes required for infection of G. mellonella

We repeated our serial infection strategy using the *S. cerevisiae* haploid deletion collection to identify genes important for survival and growth inside our host. Each strain lacks a distinct open reading frame containing a nonessential gene (Winzeler *et al.* 1999). We created eight replicate populations by pooling cultures of these strains together and passaged each population serially 10 times *in vitro* and three times *in vivo*. For each replicate population *in vivo*, we estimated the density of the yeast recovered from the host after every passage and found the density to decrease below ∼10^4^ per individual host for most replicate populations after four passages. Hence, we restricted the experiment *in vivo* to three passages to avoid a population bottleneck before the ultimate passage. Populations were subjected to barseq after the first, fifth, and 10th passage *in vitro* and after the first, second, and third passages *in vivo*. Barcodes of genes not found at >100 counts in the initial populations for each replicate were eliminated, leaving 4110 deletion mutant genotypes that were recovered at high enough abundance to quantify.

**Figure 6:**
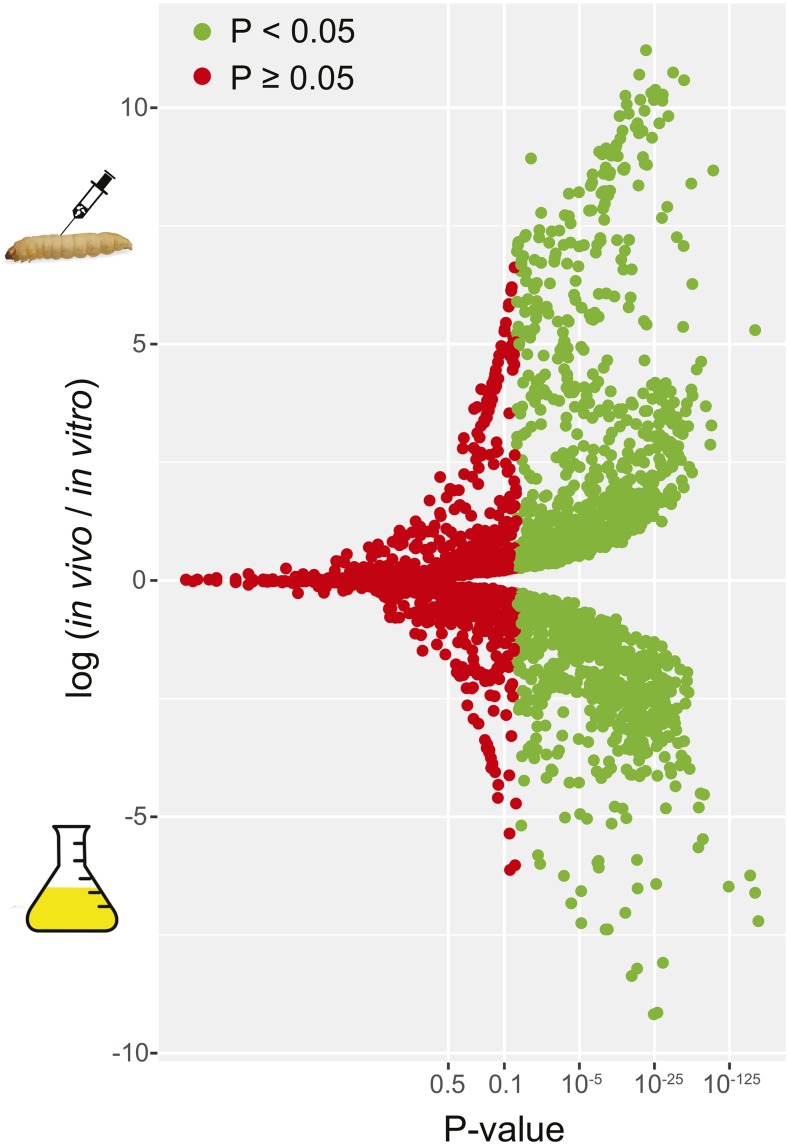
Volcano plot of mean fold change between *in vivo* *vs.* *in vitro* passages of serial passaging experiment II. Green points indicate significant *P*-values corrected using a false-discovery rate of 0.05 (Benjamini–Hochberg), and red points are nonsignificant after correction.

Most of the deletion mutants showed consistent patterns across the eight replicates. Moreover, the fitness of the mutants (*F*) was correlated across time and environments (Figure S4). Most mutants showed a consistent decrease over passages in both environments with a smaller number increasing over time (Figure S5). After the first passage, only one mutant, YJR122W, was lost from all replicate populations *in vivo*, whereas no strain disappeared completely *in vitro*. A total of ∼11% of strains (483 out of 4110) disappeared completely by the last passage (all eight replicate populations) from either environment, including 205 *in vivo* and 400 *in vitro*, out of which 122 were common between environments. We tested for differential abundance after final passages *in vitro* *vs.* *in vivo* and found 2061 genes of significantly different frequency in the two treatments (Figure 6), with 1357 of higher abundance *in vivo* *vs.* 704 higher *in vitro*. Correlation in log fold-change among genes between the two environments was highly significant (*P* = 1 × 10^−9^) but weak (*r* = 0.1).

**Figure 7:**
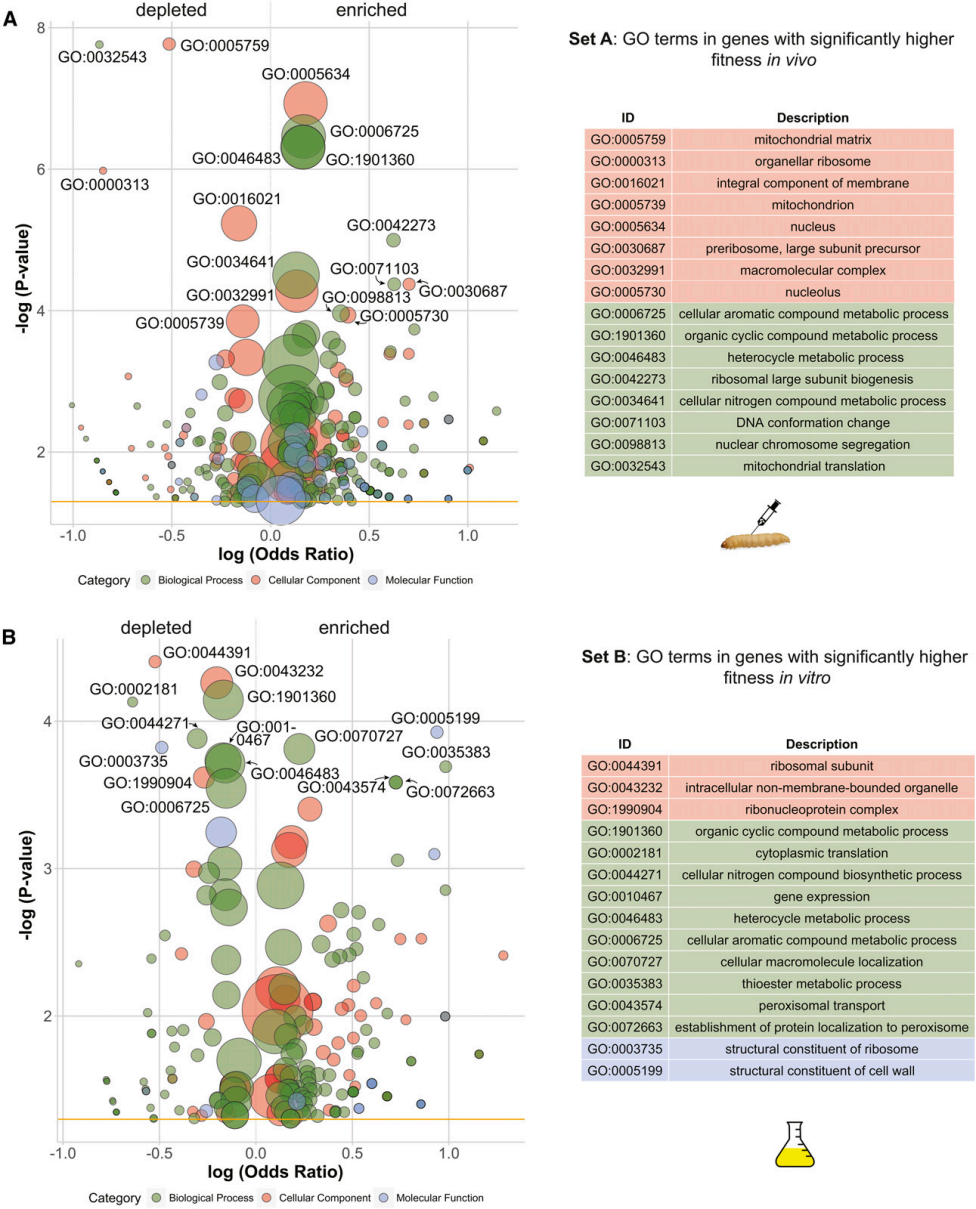
Summary of GO terms enriched and depleted in sets of deletion mutants with significantly higher fitness *in vivo* (A) or *in vitro* (B). Size of circles is proportional to the number of genes with that GO term. Values on the *x*-axis indicate log odds ratio of terms being represented by genes in the selected set *vs.* the universe of possible genes. For complete data of significant terms see Table S3.

To investigate the underlying biological differences between the differential fitness of these large sets of genes, we analyzed the sets for enrichment or depletion of relevant gene ontology (GO) terms. We analyzed GO terms in the set of genes with significantly higher fitness *in vivo* than *in vitro*, and found 49 terms significantly enriched and 13 terms significantly depleted (*P* < 0.001) in the *in vivo* set (Figure 7A). Most of the enriched terms are involved in metabolic processes such as DNA replication, translation, and chromosomal segregation, whereas the majority of the depleted terms (eight out of 13) related to mitochondria. We also analyzed the genes that had significantly higher fitness *in vitro* than *in vivo* (Figure 7B). There were 23 terms enriched in the *in vitro* set and 17 depleted (*P* < 0.001). There was an enrichment of cell wall and peroxisomal genes and a depletion of genes involved in DNA replication, translation, and repair.

We augmented this approach by considering the specific genes that had the greatest differences between *in vivo* and *in vitro* passages. The following types of patterns were considered as suggestive of candidates for pathogenicity genes: set 1, genes that were lost only from the *in vivo* passaging (positive regulators of pathogenicity); set 2, genes that were only lost from the *in vitro* passaging (negative regulators of pathogenicity); and set 3, genes that were of greatest differential fitness in the two environments. The top 10 genes (with respect to significant differences) for each of the three patterns are listed in Table 1. The functions of these sets were similar to those observed in the GO term enrichment analysis, but provide specific candidates for pathogenicity genes. Set 1 contained a cell wall integrity gene *SLT2* and six mitochondrial genes. Set 2 was enriched for DNA repair and ribosomal genes. Set 3 contained two genes involved in tyrosine metabolism as well as a gene encoding a cell wall mannoprotein, *FIT1*.

**Table 1:** Genes from serial passaging experiment II with the greatest differential fitness

Systematic Name	Standard Name	Log(Diff)	*P*-Value	Function
Set 1				
YHR030C	*SLT2*	−9.18	2.34E−25	Serine/threonine MAP kinase; cell wall integrity
YJR040W	*GEF1*	−9.14	2.30E−27	Voltage gated chloride channel
YEL024W	*RIP1*	−8.37	4.60E−16	Iron-sulfur protein of the mitochondrial cytochrome bc1 complex
YLR193C	*UPS1*	−8.21	5.77E−18	Phosphatidic acid transfer in mitochondrion
YDR116C	**MRPL1**	−7.38	6.54E−10	Mitochondrial ribosomal protein of the large subunit
YGL054C	*ERV14*	−7.38	1.69E−09	COPII-coated vesicle protein involved in vesicle formation
YPL188W	*POS5*	−7.25	6.27E−06	Mitochondrial NADH kinase
YPL262W	*FUM1*	−6.83	6.35E−05	Converts fumaric acid to L-malic acid in the TCA cycle
YBR003W	*COQ1*	−6.57	6.40E−06	Ubiquinone (coenzyme Q) biosynthesis
YDL069C	*CBS1*	−6.25	2.57E−04	Mitochondrial translational activator of the COB mRNA
Set 2				
YKL160W	*ELF1*	10.75	5.08E−38	Transcription elongation factor with a conserved zinc finger domain
YNL273W	*TOF1*	10.58	6.95E−48	Subunit of a replication-pausing checkpoint complex
YLL044W	None	10.38	3.38E−26	Dubious open reading frame
YOL041C	*NOP12*	10.28	1.45E−30	Nucleolar protein involved in pre-25S rRNA processing
YGL031C	*RPL24A*	10.24	1.20E−27	Ribosomal 60S subunit protein L24A
YER016W	*BIM1*	10.16	1.97E−25	Microtubule plus end-tracking protein
YGL078C	*DBP3*	10.15	2.28E−30	RNA-dependent ATPase involved in rRNA processing
YML121W	*GTR1*	9.82	2.05E−34	Subunit of a TORC1-stimulating GTPase complex
YLR235C	None	9.67	1.87E−28	Dubious open reading frame
YMR230W	*RPS10B*	9.37	1.79E−24	Component of the small (40S) ribosomal subunit
Set 3				
YBR166C	*TYR1*	−7.21	8.09E−233	Prephenate dehydrogenase involved in tyrosine biosynthesis
YMR057C	None	−6.61	2.58E−217	Dubious open reading frame
YDL094C	None	5.30	3.25E−217	Dubious open reading frame
YPR060C	*ARO7*	−6.24	2.23E−195	Chorismate mutase; catalyzes the conversion of chorismate to prephenate to initiate the tyrosine/phenylalanine-specific branch of aromatic amino acid biosynthesis
YJL134W	*LCB3*	−6.48	1.61E−123	Dihydrosphingosine-1-phosphate phosphatase involved in sphingolipid metabolism
YBR009C	*HHF1*	8.67	6.48E−89	Histone H4
YDR534C	*FIT1*	3.28	1.68E−85	Mannoprotein that is incorporated into the cell wall
YGL042C	None	2.87	1.39E−83	Dubious open reading frame
YIR023W	DAL81	3.69	7.07E−76	Positive regulator of genes in multiple nitrogen degradation pathways
YML048W	GSF2	−4.53	4.66E−73	Endoplasmic reticulum localized integral membrane protein

Log(Diff) represents the difference in relative abundance of a mutant in (*in vivo*/*in vitro*) passages. *P*-values are FDR corrected using the Benjamini–Hochberg method. Set 1 comprises the 10 genes of the highest differential fitness which were lost only from the *in vivo* passaging. Set 2 comprises the 10 genes of the highest differential fitness that were only lost from the *in vitro* passaging. Set 3 comprises the 10 genes that were of greatest differential fitness (ranked by *P*-value) in the two environments.

## Discussion

Here we tested the ability of a model fungus to behave as a pathogen by injecting it in large doses into living insect larvae to analyze genetic variation in pathogenicity. We found ample evidence of genetic variation in pathogenicity as evidenced by differential recovery from hosts of particular genotypes, differential mortality caused by strains, and variation at loci that explain survival in the larval environment. At the onset of this experiment, our working hypothesis was that *S. cerevisiae* would not show virulence in *G. mellonella* based on previous results showing *Candida* species to be effective at killing *G. mellonella* but not *S. cerevisiae* (Cotter *et al.* 2000). However, we found significant mortality at a similar dosage (2.5 × 10^6^ cells per larva; Figure 1) used by Cotter *et al.* (2000), and thus differences between our experiments and theirs may be due to particular yeast strains tested. This is particularly likely in this case, because Cotter *et al.* (2000) used only a single yeast strain (JJ1A), which was also auxotrophic (*arg*^−^, *thr*^−^), and threonine auxotrophs are known to have severely attenuated virulence (Kingsbury and McCusker 2010).

The results from the first passaging experiment with the wild strains showed a striking determinism in equilibrium genotype frequencies across populations, suggesting the habitats provided a selective regime that greatly outweighed genetic drift (Figure 2). We did not find that clinical strains had a higher pathogenic potential than strains from other environments. Clinical *S. cerevisiae* are associated with growth at 42° and the ability to form pseudohyphae (McCusker *et al.* 1994; Muller *et al.* 2011). However, both phenotypic and genotypic distinction of *S. cerevisiae* clinical isolates is debated (Muller and McCusker 2009; Klingberg *et al.* 2008; de Llanos *et al.* 2006). In a mouse infection model, clinical strains were in general more virulent than nonclinical strains (Clemons *et al.* 1994; Llopis *et al.* 2014). Although clinical strains were not able to survive better *in vivo* in *G. mellonella*, we found that the ability of strains to form pseudohyphae was correlated with yeast survival in *G. mellonella* (Figure 4), and this provides a commonality with *S. cerevisiae* virulence in mouse. Our genetic screens also suggested genes known to be involved in virulence in other opportunistic fungal pathogens of humans, such as the MAPK pathway and cell wall integrity genes, which have a role in pathogenicity in other opportunistic fungi (Lengeler *et al.* 2000; Xu 2000). Our hypothesis is that the diversity of *S. cerevisiae* ecological roles selects for traits such as pseudohyphal formation and cell wall integrity, which provide resistance to competitors and predators, selection on which preadapts the fungus to function as an opportunistic pathogen. The fact that this study discovered similar genetic mechanisms underlying pathogenicity in *S. cerevisiae* and other opportunistic fungi signals that there are common traits that link these fungi and gives hope that broad-ranging therapies may be discovered.

Our screen identified a number of genes associated with persistence during larval passaging, which are candidate pathogenicity factors in *S. cerevisiae* (Table 1). We identified both genes that are similar to those known from other fungal pathogen species as well as novel genes that generate hypotheses for further testing of their contribution to pathogenicity. Here we discuss three types of genes further that have important parallels with other opportunistic fungi: genes involved in cell wall integrity and filamentation, mitochondrial genes, and genes involved in tyrosine metabolism.

We identified that pseudohyphal formation, cell wall integrity, and the genes that control these processes are associated with fitness *in vivo*. First, we found that there was an association between filamentation and fitness *in vivo* (Figure 4). Filamentous growth is linked to adherence to epithelial cells which promotes biofilm formation and could prevent clearing by phagocytosis (Gale *et al.* 1998; Román *et al.* 2007). In both *C. neoformans* and *Candida* spp., cells that form pseudohyphae are able to escape phagocytosis by amoebae and macrophages, respectively (Neilson *et al.* 1978; Brunke *et al.* 2014; Wartenberg *et al.* 2014). A similar process could be occurring in the hemolymph of *G. mellonella* between yeast and phagocytic hemocytes. Second, we determined that the gene closest to the QTL on chromosome VII was *STE20*, a p21-activated signal transducing kinase that is a known pathogenicity factor in other opportunistic fungi (Calcagno *et al.* 2004; Wang *et al.* 2002), and in *S. cerevisiae* functions during mating pheromone stimulation, controls bud site selection, pseudohyphal formation, and vacuole inheritance (Gancedo 2001; Roberts and Fink 1994; Bartholomew and Hardy 2009). Unfortunately, our data were unable to conclusively determine whether the actual gene(s) underlying the effect was *STE20*, and further studies are needed. Components of MAP kinase signaling pathway in pathogenic fungi, such as Ste20, play diverse regulatory roles in multiple processes such as biofilm formation, stress response, and cell wall remodeling, which may also provide resistance to innate insect immunity (Román *et al.* 2007). In particular, cell wall integrity appears to be critical for virulence in a number of fungal pathogens (Hsu *et al.* 2015; Calcagno *et al.* 2004; Kraus *et al.* 2003). Our screen with the deletion collection identified at least two specific genes involved in cell wall integrity (*SLT2* and *FIT1*; Table 1) and also suggested that genes involved in structural constituents of the cell wall (GO: 0005199) were more likely to have low fitness *in vivo* (Figure 7). In fact, the *slt2*Δ mutant was identified as having the most significant fitness differential between *in vivo* and *in vitro*, and was lost across all *in vivo* passages. Slt2 is also a MAPK signaling gene that has a role in cell wall integrity following response to stress and virulence (Kraus *et al.* 2003; de Llanos *et al.* 2010). The Slt2 protein has previously been shown to be involved in maintaining the mannoprotein coat of the *S. cerevisiae* cell wall such that β-1,3 glucans are not exposed (Wheeler and Fink 2006). The masking of this fungal-specific antigen is likely essential for evading recognition by β-1,3 glucan binding protein in insects, which recognizes fungal glucan and activates the pathway of pathogen immobilization by melanin (Kavanagh and Reeves 2004). In summary, our genetic screens have identified proteins involved in cell wall integrity and morphogenesis that likely function in avoidance of the innate insect immune system.

The second group of genes we observed with important implication for *S. cerevisiae* pathology are genes of mitochondrial function. Our genetic screen with the yeast deletion collection identified multiple mitochondrial genes as being more vital for persistence in the host than growth in YPD (Figure 7 and Table 1). Mitochondrial genes play numerous functions beyond aerobic respiration that may be related to pathogenicity, such as control of filamentation, cell wall integrity, and response to hypoxic conditions and oxidative stress (Calderone *et al.* 2015; Ma and May 2010; Jin *et al.* 2008), and previous studies have similarly shown that respiration deficient mutants have reduced ability to survive *in vivo* using a mouse model (Goldstein and McCusker 2001). Because of the diverse functions of mitochondria, the exact mechanism(s) that links our genes to the ability to survive *in vivo* are unclear, although multiple hypotheses, such as energy efficiency and resistance to reactive oxygen species, have been proposed to explain why *S. cerevisiae* mitochondrial mutants are deficient for growth in mouse models (Goldstein and McCusker 2001).

Our final cohesive group of pathogenicity genes are those involved in cellular aromatic compound metabolism (GO: 0006725). Deletion mutants associated with this GO term were more likely to be have higher fitness *in vivo* *vs.* *in vitro*, suggesting that these compounds are less important for pathogenicity. However, two of the genes in this class were among the two genes with the greatest differential fitness disadvantage *in vivo* (*TYR1* and *ARO7*; Table 1). Both genes are part of the phenylalanine/tyrosine biosynthesis pathway and are involved in conversions to or from the tyrosine precursor prephenate. Genotypes of *S. cerevisiae* that are auxotrophic for amino acids are known to have attenuated virulence in mice, although it depends on which amino acid deficiency (Kingsbury and McCusker 2010; Goldstein and McCusker 2001). In mice, aromatic amino acids must be synthesized by the pathogen, providing target pathways for antifungal therapies (Kingsbury *et al.* 2006). It may be that the poor *tyr1*Δ and *aro7*Δ mutant fitness in *G. mellonella* derives from low hemolymph concentrations of tyrosine, phenylalanine, or the production of a toxic intermediate. However, we speculate that tyrosine *in vivo* concentration may be particularly low during the rapid production melanization response because DOPA-melanin is produced from tyrosine precursors (Figure 1A).

Beyond specific classes of genes, our approach provides insight into the architecture of genetic variation of pathogenicity through genetic mapping. Together, these data show that virulence is controlled by a large number of genes, and that there are variants segregating in natural populations that have large effect, *e.g.*, QTL. BSA identified four QTL that showed the same allele was favored in both *in vitro* and *in vivo* environments. Because *in vivo* passaging also includes a growth step in rich media, it is impossible to distinguish whether these are due to selection for or against alleles of a very general fitness effect or whether there is selection specific to the growth in rich media. BSA also identified three genomic regions in which one parental allele was favored only in the larval passaged pool, and only one of these was from the clinical parent, QTL 4 (Figure 5). The fact that loci from both parents significantly contribute to survival in the larval environment may explain the observation of heterosis we observed in the hybrid strain.

 The fact that clinical strains of *S. cerevisiae* do not form a genetic group (Muller and McCusker 2009) reinforces the notion that virulence or pathogenicity is a complex trait. At least one other study has shown that virulence in an opportunistic pathogen, *Aspergillus nidulans*, measured by inoculation of *G. mellonella* is controlled by multiple genes (Christians *et al.* 2011), which is consistent with a complex genetic architecture underlying virulence in opportunistic fungi. The complexity of virulence as a trait is magnified by the quantitative nature of traits that underlie it, such as thermotolerance, melanin production, and resistance to oxidative stress (Steinmetz *et al.* 2002; Vogan *et al.* 2016; Diezmann and Dietrich 2011).

Our study was conducted on strain pools of varying ploidy. Specifically, the wild strains of the first serial passaging were diploid, and the strains from all other experiments were haploid *S. cerevisiae* is predominantly diploid in the wild, and a large survey of 144 clinical strains has shown that clinical strains are also predominantly diploid, with the remainder roughly equally distributed among haploid, triploid, or tetraploid (Zhu *et al.* 2016). The general difference in pathogenic potential between haploid and diploid *S. cerevisiae* is uncertain, but it is known that there are clear differences in pseudohyphal formation between haploids and diploids that may lead to differences in pathogenic potential (Liu *et al.* 1996). Because of the nature of the experiments we wished to conduct, we were required to utilize haploid strains, which is the ploidy of most of the genotypes used in previous studies on pathogenicity in *S. cerevisiae* (Goldstein and McCusker 2001). Although we did not perform extensive comparisons, the fitness measured using diploids in our serial passaging experiment I (Figure 1) matched well with the coinfection experiments we conducted using pairs of haploid genotypes (Figure S2).

The increased use of invertebrate hosts to study opportunistic fungi requires pathogenicity factors to supersede host, environment, and even fungal species. To varying degrees, fungal pathogenicity factors have been correlated between mouse, *G. mellonella*, and *C. elegans* models (Desalermos *et al.* 2015; Navarro-Velasco *et al.* 2011; Brennan *et al.* 2002). In both invertebrate and vertebrate host models, common virulence factors are genes controlling filamentation, cell wall integrity, melanin production, siderophores, and intracellular signaling (Desalermos *et al.* 2015; Navarro-Velasco *et al.* 2011; Firacative *et al.* 2014; Slater *et al.* 2011). There are strong similarities between the innate immunity of insects and mammals and their similar response to fungal infection (Kavanagh and Reeves 2004). However, *G. mellonella* presents important differences from mammals such as being ectothermic, having an open circulatory system, and absence of adaptive immunity. Despite these obvious differences, common virulence factors, and higher virulence of particular clinical strains in *G. mellonella* for at least two species (Cotter *et al.* 2000; Mylonakis *et al.* 2005) suggests that it is an appropriate model host to learn insights into yeast pathogenicity. If our *in vivo* fitness analyses provide a good model for understanding pathogenicity genes, then the pathogenicity of genotypes should be correlated regardless of host. Comparison of our results with known pathogenicity factors revealed that *SSD1* (Wheeler *et al.* 2003), *MIP1* (Goldstein and McCusker 2001), and *THR1* and *THR4* (Kingsbury and McCusker 2010) deletion mutants showed similar results with deletion mutants having significantly lower pathogenicity. In contrast, we found the opposite pattern with *ADE2* and *ADE4* mutants, which are severely attenuated in pathogenicity in mouse (Goldstein and McCusker 2001), but which had a higher relative fitness in *G. mellonella* than *in vitro*. More recently, there has been development of a nematode pathogenicity model for *S. cerevisiae* using the host *Caenorhabditis elegans* (Jain *et al.* 2009). The three genes involved in resistance to reactive oxygen species that were found by the authors to be required to trigger a virulence phenotype (*SOD1*, *YAP1*, *YAP2*) were not observed to be detrimental to fitness *in vivo* in *G. mellonella*. Instead, *SOD1* deletion mutants had very low fitness in both habitats, *YAP2* had slightly higher fitness *in vivo*, and *YAP1* had a much higher relative fitness *in vivo* (41st highest of 4110). Major differences between our results and the other model hosts are not only to do with the experimental system, but also the genome-wide approach that we used, allowing us to find the genes of highest fitness effect rather than choosing candidate genes.

Our experimental system also imposed limitations due to the *in vivo* passaging strategy. In between each round of injection, pools were grown overnight in YPD to generate enough cells for the next round of injection. This was dictated by the reduction of the initial population size to ∼1% right after injection, presumably due to the action of the innate insect immunity. Both the bottlenecks imposed by the host and low spore germination led to a lower population size in our BSA that limited the mapping resolution we were able to achieve. Indeed, the mapping resolution does not allow us to identify specific genes underlying the QTL. Growth of the populations in YPD in between passages effectively creates a constantly changing environment with somewhat unpredictable outcomes through selection: antagonistic pleiotropy may limit the efficacy of selection, genotypes with higher plasticity may be favored, or balancing selection and heterosis may be favored. This latter hypothesis is particularly interesting with respect to our observation that the wild strain that was a laboratory-created hybrid with high heterozygosity was the clear winner in each of the eight replicate *in vivo* passages of serial passaging experiment I. The fact that one of the parents was a clinical strain and the other highly fit in the YPD passages may suggest that the hybrid has heterozygote advantage by combining alleles that perform well *in vivo* and *in vitro*, which was experienced during the larval passaging. There is an uncertain but significant association with clinical strains of *S. cerevisiae* and heterozygosity, with clinical strains showing roughly 50% more heterozygosity than strains from other habitats (Muller and McCusker 2009). This could possibly reflect fitness tradeoffs of mutations that provide an advantage inside the host, but whose expression bear a cost outside the host. Such fitness tradeoffs are well known across a number of yeast traits (Sunshine *et al.* 2015; Sellis *et al.* 2016).

In this study we have leveraged the tools of yeast genetics to study pathogenicity across the genome to identify a large number of candidate genes involved in survival inside an insect host. The data suggest that the host/fungus combination is an adequate model for studying pathogenicity properties of opportunistic fungi because the identification of genes in our screens overlaps with well-known virulence factors in other fungi. The approaches we employed could be readily extended to other species of fungi and other invertebrate hosts. Because environmental opportunistic fungi generally do not transmit from host to host, our passaging regime does not contradict what is observed in nature and allows persistent selection to identify alleles that function as pathogenicity factors. The most significant factors we uncovered generally point to mechanisms that would lead to resistance or evasion of innate immunity. This seems logical, given that 99% of cells were cleared immediately after injection and the strong melanization response by the host (Figure 1A). Because commonalities are found between virulence of environmental fungi in insects, mammals, and even protists, the data suggest that the primary virulence factors function at the level of innate immunity. As related pathogenicity mechanisms are detected across fungal species, they provide clues to development of novel therapies.

## Supplementary Material

Supplemental material is available online at www.g3journal.org/lookup/suppl/doi:10.1534/g3.117.300245/-/DC1.
