# Supplementary material for: Genome-Wide Screen for Saccharomyces cerevisiae Genes Contributing to Opportunistic Pathogenicity in an Invertebrate Model Host
Source: G3 (Bethesda). 2017 Nov 9;8(1):63–78. doi: 10.1534/g3.117.300245 (PMC5765367; doi:10.1534/g3.117.300245)
Supplement: Supplementary file 1 [file 63FigureS1.pdf]

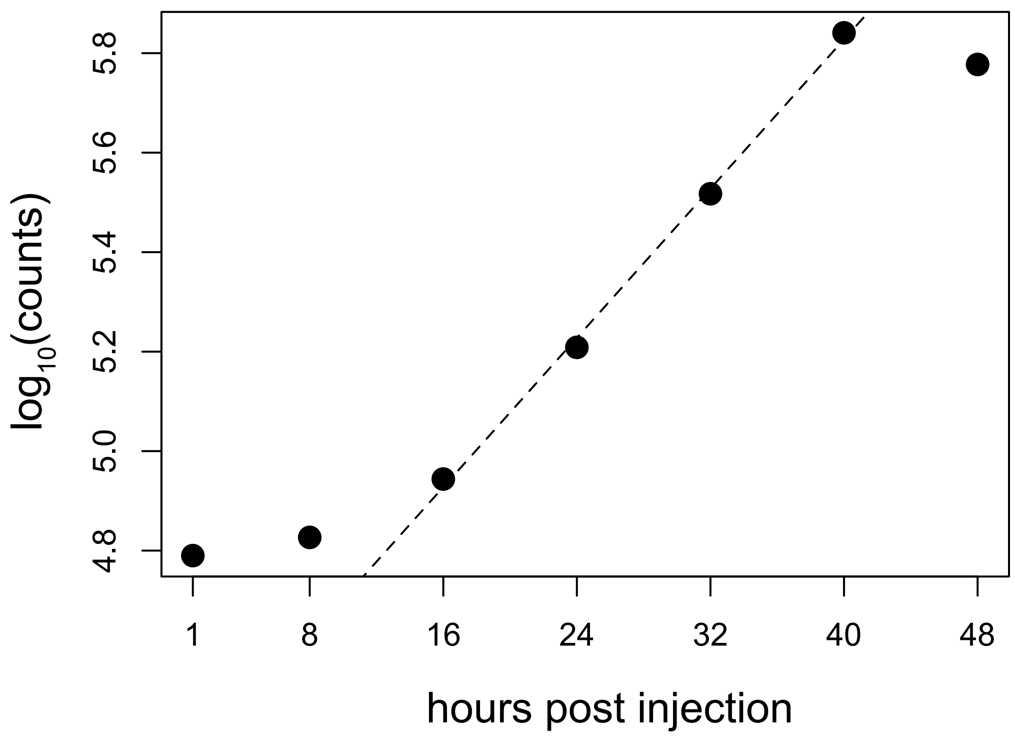

Figure S1. Estimation of *in vivo* growth rates from the log-linear portion of the cell counts obtained after injection of *G. mellonella* larvae with strain SSP245 (322134S background). Counts reflect estimated number of cells per larval individual. Estimated doubling time was 8.0 hours. See materials and methods for additional details of the experiment.
