# Supplementary material for: Genome-Wide Screen for Saccharomyces cerevisiae Genes Contributing to Opportunistic Pathogenicity in an Invertebrate Model Host
Source: G3 (Bethesda). 2017 Nov 9;8(1):63–78. doi: 10.1534/g3.117.300245 (PMC5765367; doi:10.1534/g3.117.300245)
Supplement: Supplementary file 2 [file 63FigureS2.pdf]

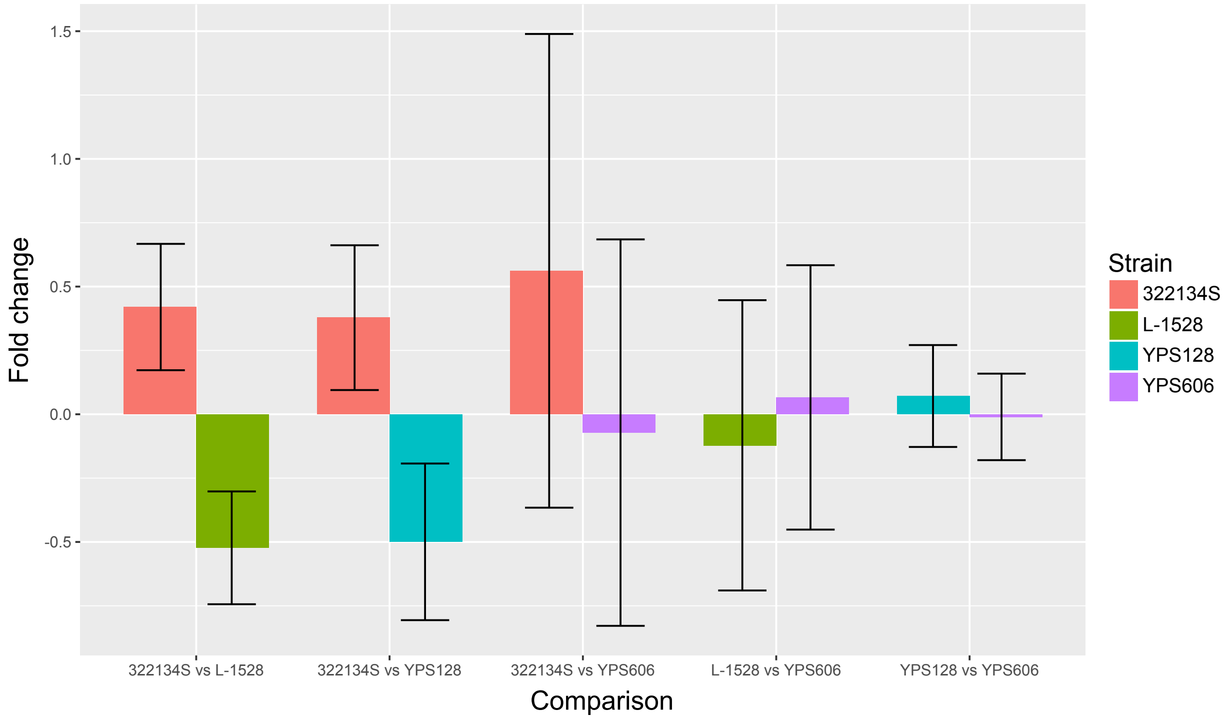

Figure S2. Relative recovery of strains following co-injection. Fold change reflects the change in frequency in the recovered population relative to the original frequency. Error bars indicate +/- one standard deviation. The first two comparisons show significantly different recovery, $t(6) = 5.7$, $P = 0.001$) and $t(6) = 4.2$, $P = 0.006$, respectively. The other comparisons were non-significant.
