# Supplementary material for: Genome-Wide Screen for Saccharomyces cerevisiae Genes Contributing to Opportunistic Pathogenicity in an Invertebrate Model Host
Source: G3 (Bethesda). 2017 Nov 9;8(1):63–78. doi: 10.1534/g3.117.300245 (PMC5765367; doi:10.1534/g3.117.300245)
Supplement: Supplementary file 3 [file 63FigureS3.pdf]

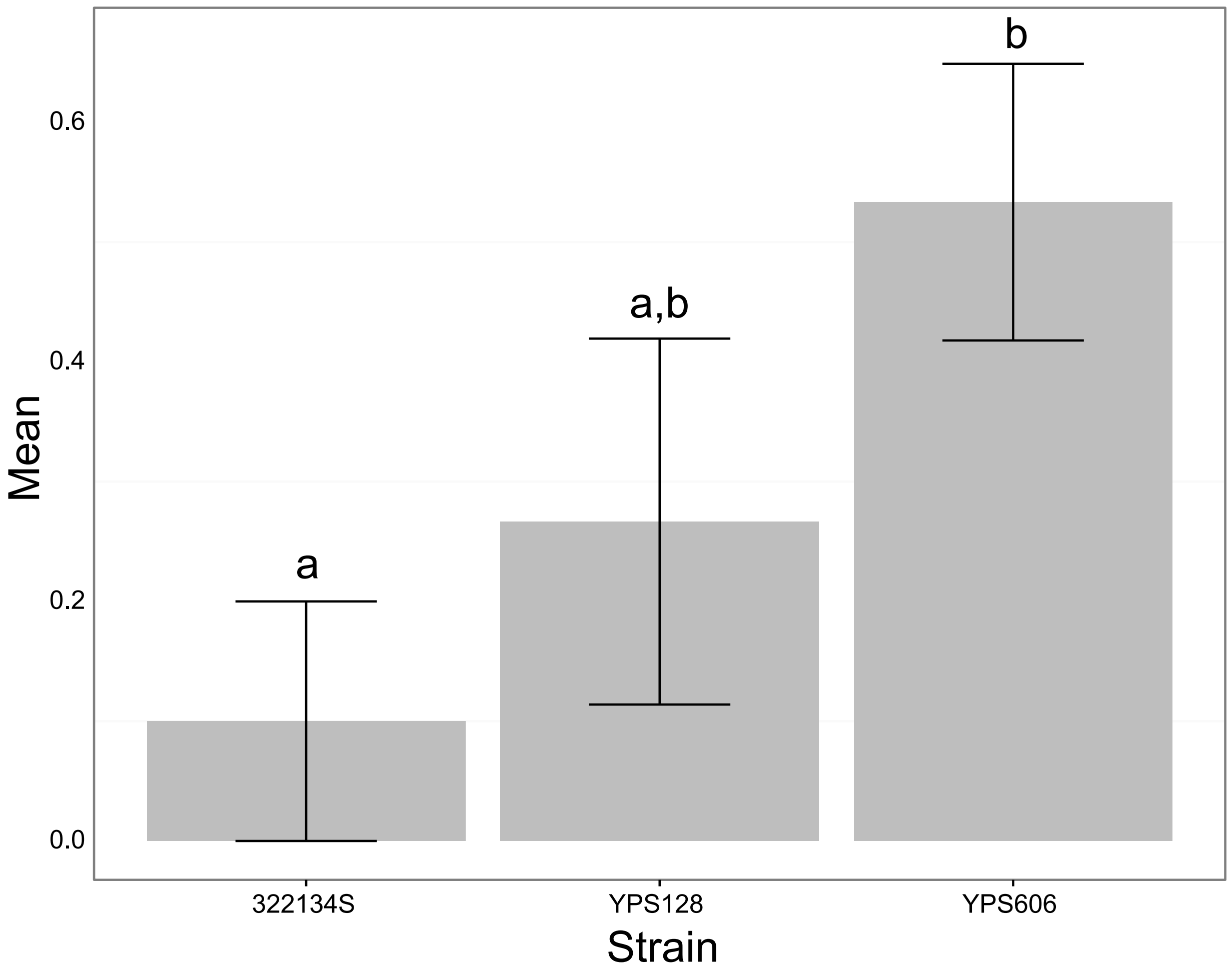

Figure S3. Percent survivorship of cohorts of larvae after inoculation with single strains. Shown are survival percentages averaged over three cohorts of 10 larvae, measured 7 days post-injection. Error bars indicate standard deviations, and shared letters (a,b) indicate comparisons that were not statistically different (Tukey's HSD, P>0.05).
