# Supplementary material for: Genome-Wide Screen for Saccharomyces cerevisiae Genes Contributing to Opportunistic Pathogenicity in an Invertebrate Model Host
Source: G3 (Bethesda). 2017 Nov 9;8(1):63–78. doi: 10.1534/g3.117.300245 (PMC5765367; doi:10.1534/g3.117.300245)
Supplement: Supplementary file 4 [file 63FigureS4.pdf]

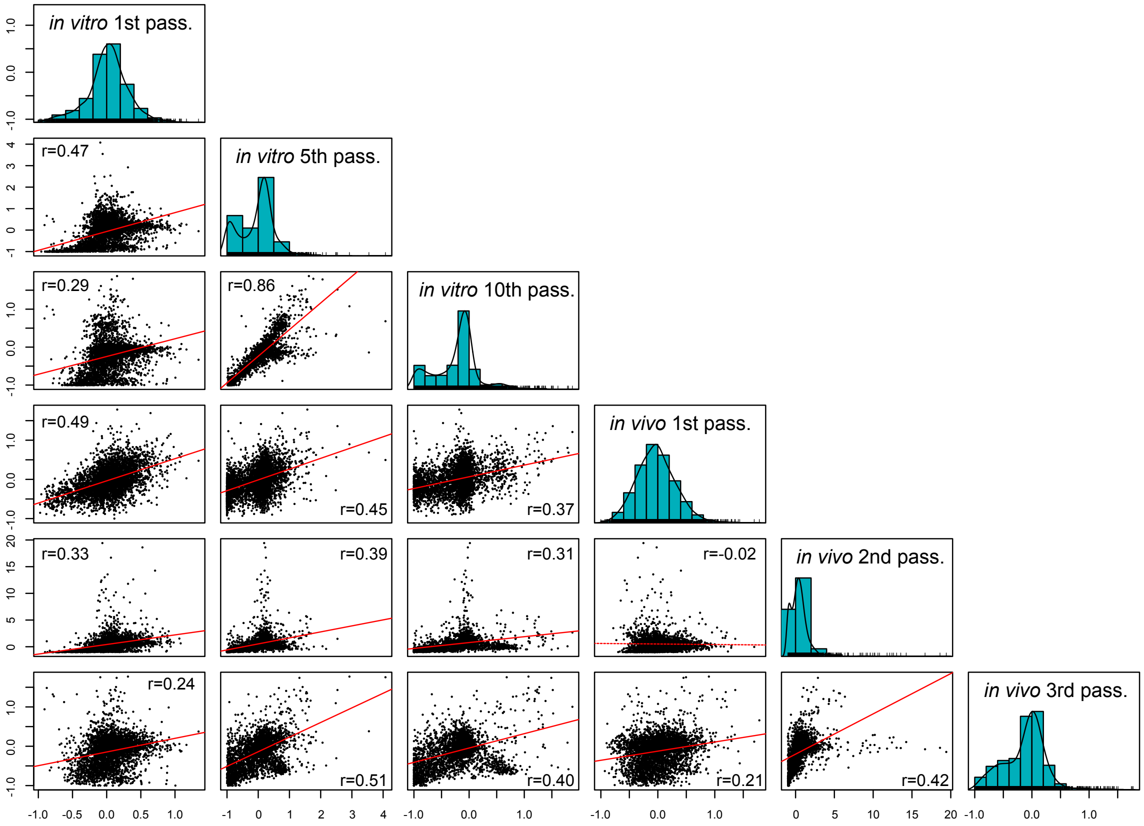

Figure S4. Correlation of fold change (*F*) between *in vitro* and *in vivo* passages of serial passaging experiment II with the deletion collection. Each point represents the *F* value of a single gene (deletion mutant). Along the diagonal are shown histograms of the distribution of *F* values. Red lines indicate linear regression between the two variables. All correlations were highly significant P< 10^{-10}, except between *in vivo* 1st and 2nd passages, which was not significant. Genes YBR101C and YDR475C_2 were high performing outliers that were removed from the plots to assist with visualization.
