# Supplementary material for: Genome-Wide Screen for Saccharomyces cerevisiae Genes Contributing to Opportunistic Pathogenicity in an Invertebrate Model Host
Source: G3 (Bethesda). 2017 Nov 9;8(1):63–78. doi: 10.1534/g3.117.300245 (PMC5765367; doi:10.1534/g3.117.300245)
Supplement: Supplementary file 5 [file 63FigureS5.pdf]

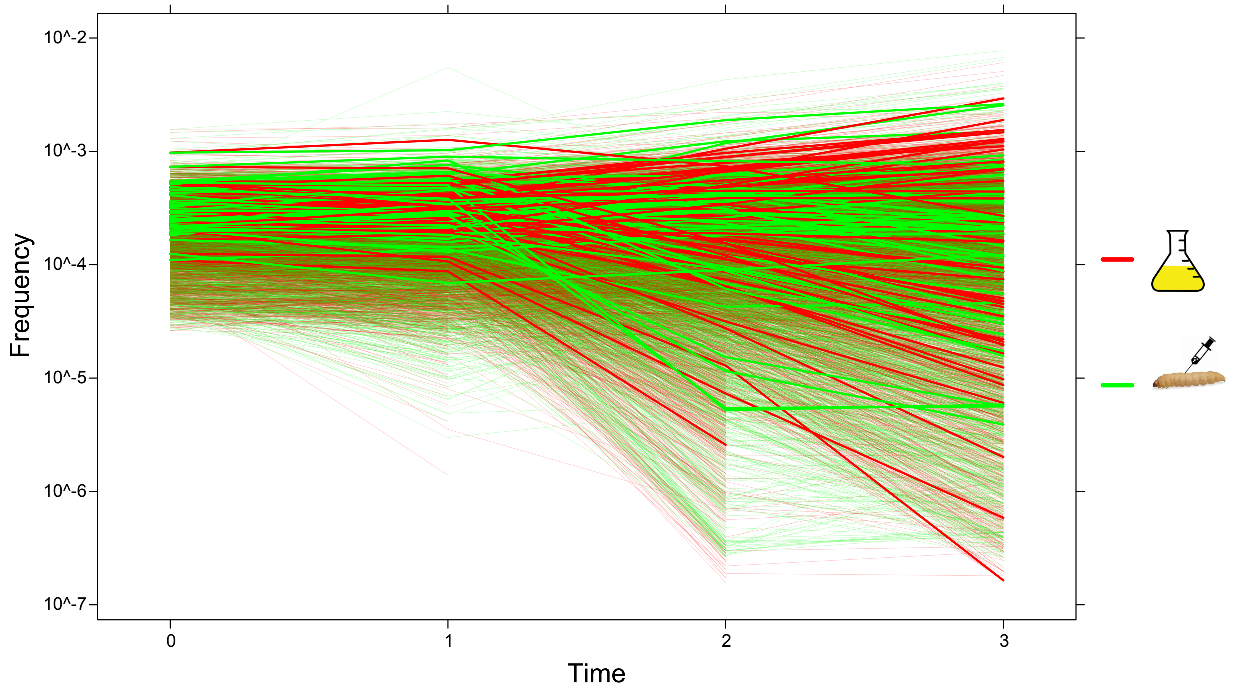

Figure S5. Change in relative frequency of 4110 deletion mutants in the two environments over passages. Time points 1, 2, and 3 for *in vitro* correspond with passages 1, 5, and 10, while those of *in vivo* are passages 1, 2, and 3. Green lines indicate means of 8 replicate *in vivo* passages, and red lines are means of 8 replicate *in vitro* passages. Lines in bold indicate the 50 genes with the highest differential fitness, measured by FDR corrected P-values from exact tests.
