# Supplementary material for: Genome-Wide Screen for Saccharomyces cerevisiae Genes Contributing to Opportunistic Pathogenicity in an Invertebrate Model Host
Source: G3 (Bethesda). 2017 Nov 9;8(1):63–78. doi: 10.1534/g3.117.300245 (PMC5765367; doi:10.1534/g3.117.300245)
Supplement: Supplementary file 6 [file 63TableS1.pdf]

Table S1. Strains used in experiments.

Strain number	Genetic background	Experiment(s)	Reference/ Construction
YPW3	Y03-GFP: *MATα, leu2Δ0, ura3Δ0, CAN1S*	Virulence estimates	(Gruber et al. 2012)
SSP96	YPS128: *ho::g418*, *MATα*	Virulence estimates; Pairwise competitions	This study
SSP104	YPS606: *ho::g418*, *MATα*	Virulence estimates; Pairwise competitions	This study
SSP243	YPS606: *ho::NAT*, *MATα*	Virulence estimates; Pairwise competitions; Bulk segregant analysis parent	(Maclean et al. 2017)
SSP245	322134S: *ho::NAT*, *MATα*	Virulence estimates; Pairwise competitions	(Maclean et al. 2017)
SSP246	YPS128: *ho::NAT*, *MATα*	Pairwise competitions	(Maclean et al. 2017)
SSP251	L-1528: *ho::g418*, *MATα*	Pairwise competitions	(Maclean et al. 2017)
SSP250	L-1528: *ho::NAT*, *MATα*	Pairwise competitions	(Maclean et al. 2017)
SSP253	322134S: *ho::g418*, *MATα*	Virulence estimates; Generation time estimation; Pairwise competitions	(Maclean et al. 2017)
SSP245	322134S: *ho::NAT*, *MATα*	Virulence estimates; Bulk segregant analysis parent	(Maclean et al. 2017)
SSP264	YPS128: *ho::g418*, *MATa*-fasterMT-RFP-hyg	Bulk segregant analysis parent	This study
SSP267	Diploid progeny of a cross between SP243 and SSP264	Bulk segregant analysis	This study
SSP272	Diploid progeny of a cross between SSP245 and SSP264	Bulk segregant analysis	This study

References:

Gruber, J.D., K. Vogel, G. Kalay, and P.J. Wittkopp, 2012 Contrasting properties of gene-specific regulatory, coding, and copy number mutations in *Saccharomyces cerevisiae*: Frequency, effects, and dominance. *PLoS Genetics* 8 (2):11.

Maclean, C.J., B.P.H. Metzger, J.-R. Yang, W.-C. Ho, B. Moyers *et al.*, 2017 Deciphering the genic basis of yeast fitness variation by simultaneous forward and reverse genetics. *Molecular Biology and Evolution* 34:2486-2502.
