# Supplementary material for: Genome-Wide Screen for Saccharomyces cerevisiae Genes Contributing to Opportunistic Pathogenicity in an Invertebrate Model Host
Source: G3 (Bethesda). 2017 Nov 9;8(1):63–78. doi: 10.1534/g3.117.300245 (PMC5765367; doi:10.1534/g3.117.300245)
Supplement: Supplementary file 7 [file 63TableS2.pdf]

Table S2. Details of the genetic background of wild strains used for passaging experiment I. Clade assignments and the procedure for integration of the barcode at the *HO* locus are described in (Maclean et al. 2017). * Isolate SSP81 is heterozygous at the barcode locus.

Strain	Geographic origin	Habitat	Clade	Uptag barcode
273614N	UK	Clinical: fecal	Other/Mosaics	GCTATATGTATCCAGAGTGG
SSP81*	UK/USA	Hybrid: Clinical/nature	Other/Mosaics x North American	TAGTCTCGTACAGTCGGCTG CCGGGTATGTTACTAGAGTT
CLIB154	Russia	Fermentation: wine	Wine/European	CCCAGATATAGAGCGATAGG
CLIB294	France	Fermentation: distillery	Wine/European	TGACTAGACTGACCTCATTG
CLIB413	China	Fermentation: rice	Sake	CCGTGGATCAAATCAGTCAA
CLIB483	France	Fermentation: cider	Wine/European	AAGCCCTCTTCGATGATTGT
DBVPG1106	Australia	Fermentation: grapes	Wine/European	GTATGACACTACCAGACAGC
DBVPG1399	Netherlands	Fermentation: grape must	Wine/European	ATATGATTTACCAGCGCGGG
DBVPG1853	Ethiopia	Fermentation: white tecc	Wine/European	GACCTGGCGATTGTCACGTT
DBVPG3591	Unknown	Nature: cocoa beans	Wine/European	CCCGCTTTCAAATGTGCTAA
DBVPG4651	Italy	Nature: truffle	Wine/European	TACTTTCTGCGAGGGACGCT
DBVPG6040	Netherlands	Fermentation: fruit juice	Other/Mosaics	TTCTTTGCAGTCGCCCAGGT
DBVPG6765	Unknown	Unknown	Wine/European	CCGAGCTATTTCATGGCATT
EM93	USA	Nature: rotting fig	Laboratory	ATTGGCTTCATGGCCCGGTT
I14	Italy	Nature: vineyard soil	Wine/European	TGCCATTCGGAACTAGGCTC
K12	Japan	Fermentation: sake	Sake	GCCTGTCTATAACTAGCTTC
L-1374	Chile	Fermentation: wine	Wine/European	GTTACCTACTTCTACACGGT
L-1528	Chile	Fermentation: wine	Wine/European	GAAGCATAAGCATGTGTGAC
M22	Italy	Fermentation: wine	Wine/European	CCCGGCATAGTAACAGATAC
NCYC361	Ireland	Fermentation: beer spoilage strain	Wine/European	AGGGCTGCTTCAGTCCTCTT
S288c	California	Laboratory: rotting fig	Laboratory	AGTTACATCCCATGCGGTCG
T73	Spain	Fermentation: wine	Wine/European	TTATGGTCGCCGCCAGACTT
UWOPS03-461.4	Malaysia	Nature: nectar, Bertam palm	Malaysian	ATATACCGCTAGGCGACTGT
UWOPS05-217.3	Malaysia	Nature: Nectar, Bertam Nature: palm	Malaysian	TGGACTGTCAGACTCGCGTT

UWOPS83-787.3	Bahamas	Nature: fruit, *Opuntia stricta*	North American	CATTACGGGTGACCAGTGAT
UWOPS87-2421	Hawaii	Nature: cladode, *Opuntia megacantha*	North American	ATATAACGCTCATGTCCCGT
WE372	South Africa	Fermentation: wine	Wine/European	TGATTGCAGACATATCCTCC
Y55	France	Fermentation: wine	Other/Mosaics	ATTGTAGGTCACGCGCCCAT
Y9J	Japan	Fermentation: wine	Wine/European	CCGATCTTCGCACTTAGTAT
YIIc17_E5	France	Fermentation: wine	Wine/European	TGATTGAGCCTCCCGCGTTT
YJM145	Ireland	Fermentation: beer	Other/Mosaics	ACGACTTGATTCACTGGCTT
YJM269	Austria	Fermentation: grapes	Other/Mosaics	TTATGCTACCGGAGCCGAGT
YJM320	USA	Clinical: blood	Other/Mosaics	TACTGTCTGGCATACCGGGT
YJM326	USA	Clinical: unknown	Other/Mosaics	TAGTCTTAGCCAGTACGGTG
YJM413	USA	Clinical: blood	Other/Mosaics	CTACGCCAGCGGATTTAAGG
YJM421	USA	Clinical: ascites fluid	Other/Mosaics	ATAACTCCTGGGACGACGGT
YJM434	Europe	Clinical: unknown	Wine/European	CGCTAGAGGTCATTCATACT
YJM436	Europe	Clinical: mouth	Other/Mosaics	TGCATCAACTTCTGCTAAGG
YJM454	USA	Clinical: blood	Other/Mosaics	CATCGGTTAGCAGGCCGTAT
YJM975	Italy	Clinical: vaginal	Wine/European	AGGGACCCGTTACCGGATTT
YJM978	Italy	Clinical: vaginal	Wine/European	TTCACTGTGGGTAAGCTGAT
YPS128	USA	Nature: soil under oak	North American	CGAGAGCGTTTCATATTGGT
YPS606	USA	Nature: oak bark	North American	CCGGGTATGTTACTAGAGTT

References:

Maclean, C.J., B.P.H. Metzger, J.-R. Yang, W.-C. Ho, B. Moyers *et al.*, 2017 Deciphering the genic basis of yeast fitness variation by simultaneous forward and reverse genetics. *Molecular Biology and Evolution* 34:2486-2502.
