# Supplementary material for: Genome-Wide Screen for Saccharomyces cerevisiae Genes Contributing to Opportunistic Pathogenicity in an Invertebrate Model Host
Source: G3 (Bethesda). 2017 Nov 9;8(1):63–78. doi: 10.1534/g3.117.300245 (PMC5765367; doi:10.1534/g3.117.300245)
Supplement: Supplementary file 8 [file 63TableS3.pdf]

Table S3. Significantly (P<0.05) enriched or depleted GO terms as plotted in Figure 7 calculated using GOstats. Counts are observed numbers of genes associated with the given Term, Size is the number of genes associated with that term in the universe of possible genes (the 4110 mutants), and ExpCount, Log (OddsRatio), and P-values are calculated from the size of the selected gene set. Genes are ranked by increasing P-value. GO terms with Log (OddsRatio) less than zero are depleted in the Set, and those greater than zero are enriched in the set. Set A = genes whose deletion mutants show higher fitness in *in vivo* than *in vitro* conditions. Set B = genes whose deletion mutants show higher fitness in *in vitro* than *in vivo* conditions.

A). GO terms from genes whose deletion mutants show higher fitness in *in vivo* than *in vitro* conditions.

Name-space	GOMFID	P-value	Log (OddsRatio)	ExpCount	Count	Size	Term
BP	GO:0032543	1.734E-08	-0.867	25.50	5	76	mitochondrial translation
BP	GO:0006725	3.449E-07	0.168	339.63	404	1015	cellular aromatic compound metabolic process
BP	GO:1901360	4.863E-07	0.164	349.00	413	1043	organic cyclic compound metabolic process
BP	GO:0046483	4.887E-07	0.166	337.63	401	1009	heterocycle metabolic process
BP	GO:0042273	1.013E-05	0.623	13.38	27	40	ribosomal large subunit biogenesis
BP	GO:0017004	1.591E-05	Undef	9.03	0	27	cytochrome complex assembly
BP	GO:0034641	3.162E-05	0.129	406.56	461	1215	cellular nitrogen compound metabolic process
BP	GO:0071103	4.240E-05	0.627	11.35	23	34	DNA conformation change
BP	GO:0098813	1.097E-04	0.357	29.78	47	89	nuclear chromosome segregation
BP	GO:0031497	1.845E-04	0.729	7.36	16	22	chromatin assembly
BP	GO:0048519	2.173E-04	0.180	116.59	147	349	negative regulation of biological process
BP	GO:0034654	2.566E-04	0.148	180.02	216	538	nucleobase-containing compound biosynthetic process
BP	GO:0002181	2.618E-04	0.308	35.47	53	106	cytoplasmic translation
BP	GO:0006913	3.267E-04	0.338	28.44	44	85	nucleocytoplasmic transport
BP	GO:0000723	3.803E-04	0.608	8.99	18	27	telomere maintenance
BP	GO:0006189	4.643E-04	Undef	2.34	7	7	'de novo' IMP biosynthetic process
BP	GO:0051276	4.945E-04	0.295	34.48	51	105	chromosome organization
BP	GO:0044238	5.451E-04	0.101	645.14	692	1928	primary metabolic process
BP	GO:0071166	8.429E-04	0.381	19.07	31	57	ribonucleoprotein complex localization
BP	GO:0019725	1.024E-03	-0.257	52.87	35	158	cellular homeostasis
BP	GO:0065004	1.292E-03	0.429	14.05	24	42	protein-DNA complex assembly
BP	GO:0016458	1.328E-03	0.281	32.40	47	97	gene silencing

BP	GO:0045814	1.382E-03	0.280	32.46	47	97	negative regulation of gene expression, epigenetic
BP	GO:1903321	1.392E-03	Undef	2.01	6	6	negative regulation of protein modification by small protein conjugation or removal
BP	GO:0033617	1.452E-03	Undef	5.35	0	16	mitochondrial respiratory chain complex IV assembly
BP	GO:0016070	1.620E-03	0.134	154.35	183	471	RNA metabolic process
BP	GO:0007064	1.642E-03	0.826	4.34	10	13	mitotic sister chromatid cohesion
BP	GO:0009987	1.694E-03	0.106	910.49	947	2721	cellular process
BP	GO:0031324	1.824E-03	0.172	86.00	108	257	negative regulation of cellular metabolic process
BP	GO:0000722	1.903E-03	0.682	5.69	12	17	telomere maintenance via recombination
BP	GO:0051172	2.055E-03	0.184	71.94	92	215	negative regulation of nitrogen compound metabolic process
BP	GO:0044249	2.168E-03	0.094	380.73	419	1140	cellular biosynthetic process
BP	GO:0006896	2.178E-03	-1.006	7.03	1	21	Golgi to vacuole transport
BP	GO:0000280	2.183E-03	0.249	37.22	52	112	nuclear division
BP	GO:0000002	2.236E-03	-0.675	10.37	3	31	mitochondrial genome maintenance
BP	GO:0044085	2.481E-03	0.120	182.70	212	546	cellular component biogenesis
BP	GO:0042276	2.631E-03	1.146	2.68	7	8	error-prone translesion synthesis
BP	GO:0009144	2.794E-03	-0.406	20.08	10	60	purine nucleoside triphosphate metabolic process
BP	GO:0048878	2.828E-03	-0.229	53.20	37	159	chemical homeostasis
BP	GO:0019219	3.231E-03	0.124	157.26	184	471	regulation of nucleobase-containing compound metabolic process
BP	GO:0033047	3.248E-03	0.428	11.71	20	35	regulation of mitotic sister chromatid segregation
BP	GO:0051304	3.248E-03	0.428	11.71	20	35	chromosome separation
BP	GO:0009890	3.389E-03	0.173	72.95	92	218	negative regulation of biosynthetic process
BP	GO:0046040	3.458E-03	0.903	3.35	8	10	IMP metabolic process
BP	GO:0010467	3.543E-03	0.097	287.77	321	860	gene expression
BP	GO:0045002	3.917E-03	0.778	4.02	9	12	double-strand break repair via single-strand annealing
BP	GO:0048308	4.035E-03	-0.525	12.72	5	38	organelle inheritance
BP	GO:0006265	4.172E-03	Undef	1.67	5	5	DNA topological change
BP	GO:0006336	4.172E-03	Undef	1.67	5	5	DNA replication-independent nucleosome assembly
BP	GO:0030491	4.172E-03	Undef	1.67	5	5	heteroduplex formation
BP	GO:0050657	4.385E-03	0.292	23.09	34	69	nucleic acid transport

BP	GO:0051983	4.448E-03	0.395	12.72	21	38	regulation of chromosome segregation
BP	GO:0006405	4.483E-03	0.361	15.06	24	45	RNA export from nucleus
BP	GO:0051236	5.013E-03	0.291	22.42	33	67	establishment of RNA localization
BP	GO:0006873	5.578E-03	-0.258	36.47	24	109	cellular ion homeostasis
BP	GO:0048193	6.465E-03	-0.263	33.80	22	101	Golgi vesicle transport
BP	GO:0043039	6.473E-03	-0.935	6.02	1	18	tRNA aminoacylation
BP	GO:0009199	6.924E-03	-0.355	20.08	11	60	ribonucleoside triphosphate metabolic process
BP	GO:0000447	6.960E-03	1.079	2.34	6	7	endonucleolytic cleavage in ITS1 to separate SSU-rRNA from 5.8S rRNA and LSU-rRNA from tricistronic rRNA transcript (SSU-rRNA, 5.8S rRNA, LSU-rRNA)
BP	GO:0006277	6.960E-03	1.079	2.34	6	7	DNA amplification
BP	GO:0046034	7.155E-03	-0.369	18.74	10	56	ATP metabolic process
BP	GO:0030490	7.323E-03	0.419	10.04	17	30	maturation of SSU-rRNA
BP	GO:0000041	7.329E-03	-0.459	13.38	6	40	transition metal ion transport
BP	GO:0090304	7.351E-03	0.189	47.71	62	150	nucleic acid metabolic process
BP	GO:1903507	7.357E-03	0.175	57.55	73	172	negative regulation of nucleic acid-templated transcription
BP	GO:0006637	7.463E-03	Undef	4.02	0	12	acyl-CoA metabolic process
BP	GO:0009059	7.510E-03	0.088	284.09	314	849	macromolecule biosynthetic process
BP	GO:0045184	7.534E-03	-0.132	118.79	98	355	establishment of protein localization
BP	GO:0009311	7.768E-03	-0.608	9.03	3	27	oligosaccharide metabolic process
BP	GO:0042254	7.986E-03	0.394	10.84	18	33	ribosome biogenesis
BP	GO:2000113	7.990E-03	0.160	67.59	84	202	negative regulation of cellular macromolecule biosynthetic process
BP	GO:0000460	8.139E-03	0.565	5.69	11	17	maturation of 5.8S rRNA
BP	GO:0046148	8.139E-03	0.565	5.69	11	17	pigment biosynthetic process
BP	GO:0051174	8.599E-03	-0.278	28.44	18	85	regulation of phosphorus metabolic process
BP	GO:0051253	8.672E-03	0.170	57.89	73	173	negative regulation of RNA metabolic process
BP	GO:0034629	8.963E-03	0.727	3.68	8	11	cellular protein complex localization
BP	GO:0006355	9.421E-03	0.112	141.88	164	424	regulation of transcription, DNA-templated
BP	GO:2001141	9.421E-03	0.112	141.88	164	424	regulation of RNA biosynthetic process
BP	GO:0006348	9.536E-03	0.303	17.40	26	52	chromatin silencing at telomere

BP	GO:0006400	9.536E-03	0.303	17.40	26	52	tRNA modification
BP	GO:0033554	1.084E-02	0.109	144.22	166	431	cellular response to stress
BP	GO:0043414	1.142E-02	0.274	20.08	29	60	macromolecule methylation
BP	GO:0071428	1.179E-02	0.461	7.36	13	22	rRNA-containing ribonucleoprotein complex export from nucleus
BP	GO:0006335	1.250E-02	Undef	1.34	4	4	DNA replication-dependent nucleosome assembly
BP	GO:0016242	1.250E-02	Undef	1.34	4	4	negative regulation of macroautophagy
BP	GO:0051444	1.250E-02	Undef	1.34	4	4	negative regulation of ubiquitin-protein transferase activity
BP	GO:0060236	1.250E-02	Undef	1.34	4	4	regulation of mitotic spindle organization
BP	GO:0006325	1.272E-02	0.180	45.26	58	137	chromatin organization
BP	GO:0007032	1.321E-02	-0.880	5.35	1	16	endosome organization
BP	GO:0007266	1.321E-02	-0.880	5.35	1	16	Rho protein signal transduction
BP	GO:0009206	1.321E-02	-0.880	5.35	1	16	purine ribonucleoside triphosphate biosynthetic process
BP	GO:0033044	1.437E-02	0.303	15.39	23	46	regulation of chromosome organization
BP	GO:0007059	1.452E-02	0.532	5.28	10	16	chromosome segregation
BP	GO:0006887	1.453E-02	0.353	11.38	18	34	exocytosis
BP	GO:0033046	1.461E-02	0.399	9.03	15	27	negative regulation of sister chromatid segregation
BP	GO:0006368	1.477E-02	0.286	17.07	25	51	transcription elongation from RNA polymerase II promoter
BP	GO:0000075	1.496E-02	0.272	18.74	27	56	cell cycle checkpoint
BP	GO:0016485	1.524E-02	-0.500	9.70	4	29	protein processing
BP	GO:0006812	1.535E-02	-0.217	36.81	26	110	cation transport
BP	GO:0006364	1.544E-02	0.361	10.63	17	32	rRNA processing
BP	GO:0045333	1.548E-02	-0.278	23.76	15	71	cellular respiration
BP	GO:0034728	1.623E-02	0.358	10.68	17	32	nucleosome organization
BP	GO:0000070	1.633E-02	0.564	4.63	9	14	mitotic sister chromatid segregation
BP	GO:0030071	1.665E-02	0.356	10.71	17	32	regulation of mitotic metaphase/anaphase transition
BP	GO:0044092	1.665E-02	0.356	10.71	17	32	negative regulation of molecular function
BP	GO:0046352	1.691E-02	Undef	3.35	0	10	disaccharide catabolic process
BP	GO:0070127	1.691E-02	Undef	3.35	0	10	tRNA aminoacylation for mitochondrial protein translation
BP	GO:0098662	1.737E-02	-0.329	17.40	10	52	inorganic cation transmembrane transport
BP	GO:0006281	1.747E-02	0.171	45.01	57	135	DNA repair

BP	GO:0018193	1.763E-02	0.188	37.05	48	111	peptidyl-amino acid modification
BP	GO:0071702	1.803E-02	-0.092	191.07	169	571	organic substance transport
BP	GO:0007019	1.807E-02	0.999	2.01	5	6	microtubule depolymerization
BP	GO:0010501	1.807E-02	0.999	2.01	5	6	RNA secondary structure unwinding
BP	GO:0018202	1.807E-02	0.999	2.01	5	6	peptidyl-histidine modification
BP	GO:0030261	1.807E-02	0.999	2.01	5	6	chromosome condensation
BP	GO:0042766	1.807E-02	0.999	2.01	5	6	nucleosome mobilization
BP	GO:0042325	1.850E-02	-0.360	14.76	8	44	regulation of phosphorylation
BP	GO:0030242	1.878E-02	-0.850	5.02	1	15	pexophagy
BP	GO:0043408	1.878E-02	-0.850	5.02	1	15	regulation of MAPK cascade
BP	GO:0046434	1.878E-02	-0.850	5.02	1	15	organophosphate catabolic process
BP	GO:0090501	1.895E-02	0.415	7.70	13	23	RNA phosphodiester bond hydrolysis
BP	GO:0000463	1.906E-02	0.602	4.02	8	12	maturation of LSU-rRNA from tricistronic rRNA transcript (SSU-rRNA, 5.8S rRNA, LSU-rRNA)
BP	GO:0000731	1.906E-02	0.602	4.02	8	12	DNA synthesis involved in DNA repair
BP	GO:0031990	1.994E-02	0.777	2.68	6	8	mRNA export from nucleus in response to heat stress
BP	GO:0097549	1.994E-02	0.777	2.68	6	8	chromatin organization involved in negative regulation of transcription
BP	GO:0051346	1.996E-02	0.668	3.35	7	10	negative regulation of hydrolase activity
BP	GO:0044724	2.148E-02	-0.318	17.07	10	51	single-organism carbohydrate catabolic process
BP	GO:0007127	2.210E-02	0.245	20.08	28	60	meiosis I
BP	GO:0060255	2.337E-02	0.081	210.01	232	629	regulation of macromolecule metabolic process
BP	GO:0006301	2.448E-02	0.439	6.36	11	19	postreplication repair
BP	GO:0006875	2.494E-02	-0.250	24.09	16	72	cellular metal ion homeostasis
BP	GO:0000959	2.512E-02	Undef	3.02	0	9	mitochondrial RNA metabolic process
BP	GO:0071174	2.518E-02	0.368	8.70	14	26	mitotic spindle checkpoint
BP	GO:1902100	2.518E-02	0.368	8.70	14	26	negative regulation of metaphase/anaphase transition of cell cycle
BP	GO:2000816	2.518E-02	0.368	8.70	14	26	negative regulation of mitotic sister chromatid separation

BP	GO:0033615	2.544E-02	Undef	3.01	0	9	mitochondrial proton-transporting ATP synthase complex assembly
BP	GO:0070272	2.544E-02	Undef	3.01	0	9	proton-transporting ATP synthase complex biogenesis
BP	GO:2000045	2.544E-02	Undef	3.01	0	9	regulation of G1/S transition of mitotic cell cycle
BP	GO:0051179	2.609E-02	-0.069	326.92	302	977	localization
BP	GO:0006879	2.632E-02	-0.420	10.37	5	31	cellular iron ion homeostasis
BP	GO:0006878	2.660E-02	-0.817	4.68	1	14	cellular copper ion homeostasis
BP	GO:0042278	2.741E-02	-0.239	25.10	17	75	purine nucleoside metabolic process
BP	GO:0071427	2.748E-02	0.330	10.37	16	31	mRNA-containing ribonucleoprotein complex export from nucleus
BP	GO:0040023	2.774E-02	0.456	5.69	10	17	establishment of nucleus localization
BP	GO:0071705	2.906E-02	0.133	61.57	74	184	nitrogen compound transport
BP	GO:0051028	2.935E-02	0.251	17.07	24	51	mRNA transport
BP	GO:0042221	3.039E-02	-0.103	117.12	101	350	response to chemical
BP	GO:0043900	3.133E-02	0.477	5.02	9	15	regulation of multi-organism process
BP	GO:0050000	3.133E-02	0.477	5.02	9	15	chromosome localization
BP	GO:0009142	3.155E-02	-0.607	6.02	2	18	nucleoside triphosphate biosynthetic process
BP	GO:0045786	3.211E-02	0.229	19.74	27	59	negative regulation of cell cycle
BP	GO:0007165	3.212E-02	-0.128	73.95	61	221	signal transduction
BP	GO:0023052	3.212E-02	-0.128	73.95	61	221	signaling
BP	GO:0008652	3.220E-02	0.190	28.44	37	85	cellular amino acid biosynthetic process
BP	GO:0009119	3.241E-02	-0.220	27.10	19	81	ribonucleoside metabolic process
BP	GO:0005975	3.283E-02	-0.134	66.25	54	198	carbohydrate metabolic process
BP	GO:0051301	3.290E-02	0.146	48.18	59	144	cell division
BP	GO:0000375	3.321E-02	0.302	11.38	17	34	RNA splicing, via transesterification reactions
BP	GO:0006487	3.381E-02	-0.403	10.04	5	30	protein N-linked glycosylation
BP	GO:0034219	3.426E-02	-0.444	8.70	4	26	carbohydrate transmembrane transport
BP	GO:0000478	3.520E-02	0.505	4.35	8	13	endonucleolytic cleavage involved in rRNA processing
BP	GO:0001100	3.520E-02	0.505	4.35	8	13	negative regulation of exit from mitosis

BP	GO:0010970	3.520E-02	0.505	4.35	8	13	transport along microtubule
BP	GO:0030473	3.520E-02	0.505	4.35	8	13	nuclear migration along microtubule
BP	GO:0055085	3.528E-02	-0.115	87.67	74	261	transmembrane transport
BP	GO:0006091	3.557E-02	-0.166	42.83	33	128	generation of precursor metabolites and energy
BP	GO:0034470	3.564E-02	0.208	22.47	30	68	ncRNA processing
BP	GO:0033108	3.667E-02	-0.785	4.37	1	13	mitochondrial respiratory chain complex assembly
BP	GO:0007018	3.707E-02	Undef	1.00	3	3	microtubule-based movement
BP	GO:0000162	3.740E-02	Undef	1.00	3	3	tryptophan biosynthetic process
BP	GO:0000393	3.740E-02	Undef	1.00	3	3	spliceosomal conformational changes to generate catalytic conformation
BP	GO:0000712	3.740E-02	Undef	1.00	3	3	resolution of meiotic recombination intermediates
BP	GO:0001308	3.740E-02	Undef	1.00	3	3	negative regulation of chromatin silencing involved in replicative cell aging
BP	GO:0006272	3.740E-02	Undef	1.00	3	3	leading strand elongation
BP	GO:0006688	3.740E-02	Undef	1.00	3	3	glycosphingolipid biosynthetic process
BP	GO:0010978	3.740E-02	Undef	1.00	3	3	gene silencing involved in chronological cell aging
BP	GO:0015888	3.740E-02	Undef	1.00	3	3	thiamine transport
BP	GO:0030472	3.740E-02	Undef	1.00	3	3	mitotic spindle organization in nucleus
BP	GO:0031047	3.740E-02	Undef	1.00	3	3	gene silencing by RNA
BP	GO:0031589	3.740E-02	Undef	1.00	3	3	cell-substrate adhesion
BP	GO:0033683	3.740E-02	Undef	1.00	3	3	nucleotide-excision repair, DNA incision
BP	GO:0042435	3.740E-02	Undef	1.00	3	3	indole-containing compound biosynthetic process
BP	GO:0043709	3.740E-02	Undef	1.00	3	3	cell adhesion involved in single-species biofilm formation
BP	GO:0046084	3.740E-02	Undef	1.00	3	3	adenine biosynthetic process
BP	GO:0051436	3.740E-02	Undef	1.00	3	3	negative regulation of ubiquitin-protein ligase activity involved in mitotic cell cycle
BP	GO:0070869	3.740E-02	Undef	1.00	3	3	heterochromatin assembly involved in chromatin silencing
BP	GO:0072698	3.740E-02	Undef	1.00	3	3	protein localization to microtubule cytoskeleton
BP	GO:0090231	3.740E-02	Undef	1.00	3	3	regulation of spindle checkpoint

BP	GO:0090266	3.740E-02	Undef	1.00	3	3	regulation of mitotic cell cycle spindle assembly checkpoint
BP	GO:0090399	3.740E-02	Undef	1.00	3	3	replicative senescence
BP	GO:0090605	3.740E-02	Undef	1.00	3	3	submerged biofilm formation
BP	GO:1903088	3.740E-02	Undef	1.00	3	3	5-amino-1-ribofuranosylimidazole-4-carboxamide transmembrane transport
BP	GO:1903828	3.740E-02	Undef	1.00	3	3	negative regulation of cellular protein localization
BP	GO:0007129	3.753E-02	-0.782	4.35	1	13	synapsis
BP	GO:0010821	3.753E-02	-0.782	4.35	1	13	regulation of mitochondrion organization
BP	GO:0015986	3.753E-02	-0.782	4.35	1	13	ATP synthesis coupled proton transport
BP	GO:0042326	3.753E-02	-0.782	4.35	1	13	negative regulation of phosphorylation
BP	GO:0070071	3.753E-02	-0.782	4.35	1	13	proton-transporting two-sector ATPase complex assembly
BP	GO:1904669	3.753E-02	-0.782	4.35	1	13	ATP export
BP	GO:0015791	3.827E-02	Undef	2.68	0	8	polyol transport
BP	GO:0031098	3.827E-02	Undef	2.68	0	8	stress-activated protein kinase signaling cascade
BP	GO:0070816	3.827E-02	Undef	2.68	0	8	phosphorylation of RNA polymerase II C-terminal domain
BP	GO:0018105	3.828E-02	0.388	6.69	11	20	peptidyl-serine phosphorylation
BP	GO:0000724	3.831E-02	0.301	10.71	16	32	double-strand break repair via homologous recombination
BP	GO:0006383	3.921E-02	0.543	3.68	7	11	transcription from RNA polymerase III promoter
BP	GO:0006995	3.921E-02	0.543	3.68	7	11	cellular response to nitrogen starvation
BP	GO:0031365	3.921E-02	0.543	3.68	7	11	N-terminal protein amino acid modification
BP	GO:0070828	3.921E-02	0.543	3.68	7	11	heterochromatin organization
BP	GO:0055076	4.187E-02	-0.225	23.09	16	69	transition metal ion homeostasis
BP	GO:0071852	4.199E-02	-0.152	46.85	37	140	fungal-type cell wall organization or biogenesis
BP	GO:0015865	4.281E-02	-0.579	5.69	2	17	purine nucleotide transport
BP	GO:0007021	4.302E-02	0.601	3.01	6	9	tubulin complex assembly
BP	GO:0031125	4.302E-02	0.601	3.01	6	9	rRNA 3'-end processing
BP	GO:0043174	4.302E-02	0.601	3.01	6	9	nucleoside salvage
BP	GO:1903311	4.302E-02	0.601	3.01	6	9	regulation of mRNA metabolic process

BP	GO:2000278	4.302E-02	0.601	3.01	6	9	regulation of DNA biosynthetic process
BP	GO:0006338	4.305E-02	0.245	15.02	21	45	chromatin remodeling
BP	GO:0051651	4.320E-02	-0.385	9.70	5	29	maintenance of location in cell
BP	GO:0006576	4.407E-02	0.398	6.02	10	18	cellular biogenic amine metabolic process
BP	GO:0071472	4.407E-02	0.398	6.02	10	18	cellular response to salt stress
BP	GO:0006886	4.415E-02	-0.114	78.30	66	234	intracellular protein transport
BP	GO:0007029	4.457E-02	-0.482	7.03	3	21	endoplasmic reticulum organization
BP	GO:0045991	4.457E-02	-0.482	7.03	3	21	carbon catabolite activation of transcription
BP	GO:0016237	4.498E-02	0.277	11.71	17	35	lysosomal microautophagy
BP	GO:0000245	4.579E-02	0.902	1.67	4	5	spliceosomal complex assembly
BP	GO:0000729	4.579E-02	0.902	1.67	4	5	DNA double-strand break processing
BP	GO:0006607	4.579E-02	0.902	1.67	4	5	NLS-bearing protein import into nucleus
BP	GO:0009164	4.579E-02	0.902	1.67	4	5	nucleoside catabolic process
BP	GO:0010526	4.579E-02	0.902	1.67	4	5	negative regulation of transposition, RNA-mediated
BP	GO:0017183	4.579E-02	0.902	1.67	4	5	peptidyl-diphthamide biosynthetic process from peptidyl-histidine
BP	GO:0032071	4.579E-02	0.902	1.67	4	5	regulation of endodeoxyribonuclease activity
BP	GO:0071930	4.579E-02	0.902	1.67	4	5	negative regulation of transcription involved in G1/S transition of mitotic cell cycle
BP	GO:1900151	4.579E-02	0.902	1.67	4	5	regulation of nuclear-transcribed mRNA catabolic process, deadenylation-dependent decay
BP	GO:0010993	4.585E-02	0.698	2.34	5	7	regulation of ubiquitin homeostasis
BP	GO:0032069	4.585E-02	0.698	2.34	5	7	regulation of nuclease activity
BP	GO:0034087	4.585E-02	0.698	2.34	5	7	establishment of mitotic sister chromatid cohesion
BP	GO:0010639	4.764E-02	0.197	22.08	29	66	negative regulation of organelle organization
BP	GO:0098771	4.797E-02	-0.167	36.47	28	109	inorganic ion homeostasis
BP	GO:0000398	4.800E-02	0.340	7.68	12	23	mRNA splicing, via spliceosome
BP	GO:0006310	4.834E-02	0.154	35.47	44	106	DNA recombination

BP	GO:0051783	4.914E-02	0.175	27.44	35	82	regulation of nuclear division
BP	GO:0032270	4.973E-02	-0.231	20.41	14	61	positive regulation of cellular protein metabolic process
BP	GO:0045839	4.984E-02	0.412	5.34	9	16	negative regulation of mitotic nuclear division
CC	GO:0005759	1.702E-08	-0.513	50.86	21	152	mitochondrial matrix
CC	GO:0005634	1.174E-07	0.178	328.19	394	994	nucleus
CC	GO:0000313	1.053E-06	-0.848	19.74	4	59	organellar ribosome
CC	GO:0016021	5.854E-06	-0.159	308.18	254	921	integral component of membrane
CC	GO:0030687	4.241E-05	0.702	9.37	20	28	preribosome, large subunit precursor
CC	GO:0032991	5.296E-05	0.133	329.41	379	993	macromolecular complex
CC	GO:0005730	1.157E-04	0.392	24.36	40	73	nucleolus
CC	GO:0005739	1.412E-04	-0.141	258.32	216	772	mitochondrion
CC	GO:0005871	1.548E-04	Undef	2.68	8	8	kinesin complex
CC	GO:0005657	4.086E-04	0.701	7.03	15	21	replication fork
CC	GO:0000776	4.093E-04	0.604	9.03	18	27	kinetochore
CC	GO:0044425	4.583E-04	-0.121	308.80	268	911	membrane part
CC	GO:0031090	4.814E-04	-0.228	76.59	54	225	organelle membrane
CC	GO:0098687	6.906E-04	0.343	24.39	38	73	chromosomal region
CC	GO:0005762	8.514E-04	-0.720	11.38	3	34	mitochondrial large ribosomal subunit
CC	GO:0022625	9.556E-04	0.384	18.40	30	55	cytosolic large ribosomal subunit
CC	GO:0005740	1.720E-03	-0.179	94.36	72	282	mitochondrial envelope
CC	GO:0005783	1.885E-03	-0.151	130.63	105	389	endoplasmic reticulum
CC	GO:0030529	2.322E-03	0.152	106.45	130	319	intracellular ribonucleoprotein complex
CC	GO:0005694	3.214E-03	0.328	19.48	30	59	chromosome
CC	GO:0031298	3.458E-03	0.903	3.35	8	10	replication fork protection complex
CC	GO:0000778	3.781E-03	0.571	6.69	13	20	condensed nuclear chromosome kinetochore
CC	GO:0098562	3.917E-03	0.778	4.02	9	12	cytoplasmic side of membrane
CC	GO:0032993	4.104E-03	0.699	4.68	10	14	protein-DNA complex
CC	GO:0005763	4.513E-03	-0.960	6.36	1	19	mitochondrial small ribosomal subunit
CC	GO:0098800	4.549E-03	-0.426	16.73	8	50	inner mitochondrial membrane protein complex
CC	GO:0098573	5.784E-03	-0.441	15.06	7	45	intrinsic component of mitochondrial membrane
CC	GO:0000228	5.793E-03	0.187	53.54	69	160	nuclear chromosome
CC	GO:0099080	5.830E-03	0.367	13.72	22	41	supramolecular complex
CC	GO:0099512	5.830E-03	0.367	13.72	22	41	supramolecular fiber
CC	GO:0071004	6.960E-03	1.079	2.34	6	7	U2-type prespliceosome
CC	GO:0005886	7.171E-03	-0.151	92.68	74	275	plasma membrane
CC	GO:0000779	8.274E-03	0.427	9.37	16	28	condensed chromosome, centromeric region
CC	GO:0005623	8.558E-03	0.130	1098.54	1119	3283	cell
CC	GO:1905368	8.683E-03	-0.534	10.37	4	31	peptidase complex

CC	GO:0031305	8.875E-03	-0.704	7.36	2	22	integral component of mitochondrial inner membrane
CC	GO:0005743	9.103E-03	-0.253	33.12	22	98	mitochondrial inner membrane
CC	GO:0030136	1.153E-02	-0.518	10.04	4	30	clathrin-coated vesicle
CC	GO:0097708	1.193E-02	-0.281	25.43	16	76	intracellular vesicle
CC	GO:0000417	1.250E-02	Undef	1.34	4	4	HIR complex
CC	GO:0005742	1.250E-02	Undef	1.34	4	4	mitochondrial outer membrane translocase complex
CC	GO:0031234	1.250E-02	Undef	1.34	4	4	extrinsic component of cytoplasmic side of plasma membrane
CC	GO:0005789	1.401E-02	-0.155	70.94	56	212	endoplasmic reticulum membrane
CC	GO:0031975	1.407E-02	-0.120	117.78	99	352	envelope
CC	GO:0016023	1.442E-02	-0.273	25.10	16	75	cytoplasmic, membrane-bounded vesicle
CC	GO:0005874	1.453E-02	0.353	11.38	18	34	microtubule
CC	GO:1990351	1.524E-02	-0.500	9.70	4	29	transporter complex
CC	GO:0022627	1.572E-02	0.325	13.05	20	39	cytosolic small ribosomal subunit
CC	GO:0022626	1.680E-02	1.009	1.98	5	6	cytosolic ribosome
CC	GO:0000788	1.807E-02	0.999	2.01	5	6	nuclear nucleosome
CC	GO:0031011	1.807E-02	0.999	2.01	5	6	Ino80 complex
CC	GO:0044424	1.830E-02	0.101	918.92	939	2827	intracellular part
CC	GO:0005643	1.910E-02	0.360	10.04	16	30	nuclear pore
CC	GO:0044815	1.994E-02	0.777	2.68	6	8	DNA packaging complex
CC	GO:0030134	2.313E-02	-0.633	6.36	2	19	ER to Golgi transport vesicle
CC	GO:0098588	2.380E-02	-0.099	143.55	125	429	bounding membrane of organelle
CC	GO:0044431	2.454E-02	-0.184	41.49	31	124	Golgi apparatus part
CC	GO:0030173	2.660E-02	-0.817	4.68	1	14	integral component of Golgi membrane
CC	GO:0070210	2.660E-02	-0.817	4.68	1	14	Rpd3L-Expanded complex
CC	GO:0099023	2.660E-02	-0.817	4.68	1	14	tethring complex
CC	GO:0098827	3.373E-02	-0.505	7.36	3	22	endoplasmic reticulum subcompartment
CC	GO:0031965	3.397E-02	0.262	14.72	21	44	nuclear membrane
CC	GO:0043232	3.485E-02	0.089	135.17	152	419	intracellular non-membrane-bounded organelle
CC	GO:0005782	3.520E-02	0.505	4.35	8	13	peroxisomal matrix
CC	GO:0000110	3.740E-02	Undef	1.00	3	3	nucleotide-excision repair factor 1 complex
CC	GO:0005685	3.740E-02	Undef	1.00	3	3	U1 snRNP
CC	GO:0016281	3.740E-02	Undef	1.00	3	3	eukaryotic translation initiation factor 4F complex
CC	GO:0031105	3.740E-02	Undef	1.00	3	3	septin complex
CC	GO:0031417	3.740E-02	Undef	1.00	3	3	NatC complex
CC	GO:0033186	3.740E-02	Undef	1.00	3	3	CAF-1 complex

CC	GO:0034274	3.740E-02	Undef	1.00	3	3	Atg12-Atg5-Atg16 complex
CC	GO:0034506	3.740E-02	Undef	1.00	3	3	chromosome, centromeric core domain
CC	GO:0043505	3.740E-02	Undef	1.00	3	3	CENP-A containing nucleosome
CC	GO:0070823	3.740E-02	Undef	1.00	3	3	HDA1 complex
CC	GO:1990615	3.740E-02	Undef	1.00	3	3	Kelch-containing formin regulatory complex
CC	GO:0030119	3.753E-02	-0.782	4.35	1	13	AP-type membrane coat adaptor complex
CC	GO:0033177	3.753E-02	-0.782	4.35	1	13	proton-transporting two-sector ATPase complex, proton-transporting domain
CC	GO:0031226	3.891E-02	-0.189	32.46	24	97	intrinsic component of plasma membrane
CC	GO:0030532	3.921E-02	0.543	3.68	7	11	small nuclear ribonucleoprotein complex
CC	GO:0044452	3.921E-02	0.543	3.68	7	11	nucleolar part
CC	GO:0031310	4.302E-02	0.601	3.01	6	9	intrinsic component of vacuolar membrane
CC	GO:0032541	4.457E-02	-0.482	7.03	3	21	cortical endoplasmic reticulum
CC	GO:0016272	4.579E-02	0.902	1.67	4	5	prefoldin complex
CC	GO:0042575	4.579E-02	0.902	1.67	4	5	DNA polymerase complex
CC	GO:0071013	4.579E-02	0.902	1.67	4	5	catalytic step 2 spliceosome
CC	GO:0000785	4.638E-02	0.165	31.79	40	95	chromatin
MF	GO:0015075	5.397E-04	-0.274	52.87	34	158	ion transmembrane transporter activity
MF	GO:0022890	1.541E-03	-0.349	28.78	16	86	inorganic cation transmembrane transporter activity
MF	GO:0003777	3.458E-03	0.903	3.35	8	10	microtubule motor activity
MF	GO:0004175	4.549E-03	-0.426	16.73	8	50	endopeptidase activity
MF	GO:0008170	5.111E-03	0.465	9.03	16	27	N-methyltransferase activity
MF	GO:0003676	5.830E-03	0.136	106.98	128	325	nucleic acid binding
MF	GO:0015078	7.329E-03	-0.459	13.38	6	40	hydrogen ion transmembrane transporter activity
MF	GO:0008017	8.139E-03	0.565	5.69	11	17	microtubule binding
MF	GO:0003723	1.103E-02	0.129	96.70	115	289	RNA binding
MF	GO:0008233	1.246E-02	-0.264	27.77	18	83	peptidase activity
MF	GO:0008092	1.307E-02	0.273	19.41	28	58	cytoskeletal protein binding
MF	GO:0016741	1.436E-02	0.252	22.04	31	66	transferase activity, transferring one-carbon groups
MF	GO:0016779	1.461E-02	0.399	9.03	15	27	nucleotidyltransferase activity

MF	GO:0044877	1.487E-02	0.184	41.16	53	123	macromolecular complex binding
MF	GO:0015144	1.524E-02	-0.500	9.70	4	29	carbohydrate transmembrane transporter activity
MF	GO:0050661	1.691E-02	Undef	3.35	0	10	NADP binding
MF	GO:0008574	1.807E-02	0.999	2.01	5	6	ATP-dependent microtubule motor activity, plus-end-directed
MF	GO:0015266	1.807E-02	0.999	2.01	5	6	protein channel activity
MF	GO:0004812	1.878E-02	-0.850	5.02	1	15	aminoacyl-tRNA ligase activity
MF	GO:0016875	1.878E-02	-0.850	5.02	1	15	ligase activity, forming carbon-oxygen bonds
MF	GO:0003697	1.910E-02	0.360	10.04	16	30	single-stranded DNA binding
MF	GO:0016628	2.544E-02	Undef	3.01	0	9	oxidoreductase activity, acting on the CH-CH group of donors, NAD or NADP as acceptor
MF	GO:0008757	2.563E-02	0.253	17.73	25	53	S-adenosylmethionine-dependent methyltransferase activity
MF	GO:0046915	2.628E-02	-0.464	9.03	4	27	transition metal ion transmembrane transporter activity
MF	GO:0046982	2.774E-02	0.456	5.69	10	17	protein heterodimerization activity
MF	GO:0043566	2.893E-02	0.374	8.03	13	24	structure-specific DNA binding
MF	GO:0030554	2.911E-02	0.101	112.77	129	337	adenyl nucleotide binding
MF	GO:0022857	3.116E-02	-0.116	91.35	77	273	transmembrane transporter activity
MF	GO:0016763	3.133E-02	0.477	5.02	9	15	transferase activity, transferring pentosyl groups
MF	GO:0031491	3.133E-02	0.477	5.02	9	15	nucleosome binding
MF	GO:0005524	3.460E-02	0.097	112.43	128	336	ATP binding
MF	GO:0000384	3.740E-02	Undef	1.00	3	3	first spliceosomal transesterification activity
MF	GO:0000386	3.740E-02	Undef	1.00	3	3	second spliceosomal transesterification activity
MF	GO:0004030	3.740E-02	Undef	1.00	3	3	aldehyde dehydrogenase [NAD(P)+] activity
MF	GO:0004075	3.740E-02	Undef	1.00	3	3	biotin carboxylase activity
MF	GO:0004477	3.740E-02	Undef	1.00	3	3	methenyltetrahydrofolate cyclohydrolase activity
MF	GO:0004488	3.740E-02	Undef	1.00	3	3	methylenetetrahydrofolate dehydrogenase (NADP+) activity
MF	GO:0008902	3.740E-02	Undef	1.00	3	3	hydroxymethylpyrimidine kinase activity
MF	GO:0008972	3.740E-02	Undef	1.00	3	3	phosphomethylpyrimidine kinase activity

MF	GO:0047536	3.740E-02	Undef	1.00	3	3	2-aminoadipate transaminase activity
MF	GO:0050334	3.740E-02	Undef	1.00	3	3	thiaminase activity
MF	GO:1903089	3.740E-02	Undef	1.00	3	3	5-amino-1-ribofuranosylimidazole-4-carboxamide transporter activity
MF	GO:0001047	3.827E-02	Undef	2.68	0	8	core promoter binding
MF	GO:0008175	3.921E-02	0.543	3.68	7	11	tRNA methyltransferase activity
MF	GO:0003887	4.302E-02	0.601	3.01	6	9	DNA-directed DNA polymerase activity
MF	GO:0015295	4.320E-02	-0.385	9.70	5	29	solute:proton symporter activity
MF	GO:0016279	4.407E-02	0.398	6.02	10	18	protein-lysine N-methyltransferase activity
MF	GO:0004386	4.498E-02	0.277	11.71	17	35	helicase activity
MF	GO:0046872	4.547E-02	-0.079	166.97	150	499	metal ion binding
MF	GO:0000014	4.579E-02	0.902	1.67	4	5	single-stranded DNA endodeoxyribonuclease activity
MF	GO:0000150	4.579E-02	0.902	1.67	4	5	recombinase activity
MF	GO:0000182	4.579E-02	0.902	1.67	4	5	rDNA binding
MF	GO:0004029	4.585E-02	0.698	2.34	5	7	aldehyde dehydrogenase (NAD) activity
MF	GO:0016742	4.585E-02	0.698	2.34	5	7	hydroxymethyl-, formyl- and related transferase activity
MF	GO:0022884	4.585E-02	0.698	2.34	5	7	macromolecule transmembrane transporter activity
MF	GO:0003729	4.638E-02	0.165	31.79	40	95	mRNA binding
MF	GO:0005488	4.816E-02	0.053	506.48	530	1529	binding
MF	GO:0016616	4.823E-02	-0.273	15.73	10	47	oxidoreductase activity, acting on the CH-OH group of donors, NAD or NADP as acceptor

B). GO terms from genes whose deletion mutants show higher fitness in *in vitro* than *in vivo* conditions.

Name-space	GOMFID	P-value	Log (OddsRatio)	ExpCount	Count	Size	Term
BP	GO:1901360	7.176E-05	-0.169	181.09	142	1043	organic cyclic compound metabolic process
BP	GO:0002181	7.412E-05	-0.639	18.40	5	106	cytoplasmic translation
BP	GO:0044271	1.312E-04	-0.304	50.73	29	281	cellular nitrogen compound biosynthetic process
BP	GO:0070727	1.543E-04	0.225	56.08	81	323	cellular macromolecule localization
BP	GO:0010467	1.860E-04	-0.170	149.32	115	860	gene expression
BP	GO:0046483	1.931E-04	-0.159	175.19	139	1009	heterocycle metabolic process
BP	GO:0035383	2.035E-04	0.984	2.08	8	12	thioester metabolic process
BP	GO:0043574	2.600E-04	0.725	3.65	11	21	peroxisomal transport

BP	GO:0072663	2.600E-04	0.725	3.65	11	21	establishment of protein localization to peroxisome
BP	GO:0006725	2.855E-04	-0.154	176.23	141	1015	cellular aromatic compound metabolic process
BP	GO:0019319	8.766E-04	0.734	2.95	9	17	hexose biosynthetic process
BP	GO:0009249	9.017E-04	Undef	0.69	4	4	protein lipoylation
BP	GO:0006807	9.269E-04	-0.161	128.87	101	719	nitrogen compound metabolic process
BP	GO:0043603	1.069E-03	-0.243	53.13	34	306	cellular amide metabolic process
BP	GO:0051179	1.302E-03	0.128	169.63	201	977	localization
BP	GO:0006111	1.400E-03	0.982	1.56	6	9	regulation of gluconeogenesis
BP	GO:0016070	1.502E-03	-0.159	113.97	88	653	RNA metabolic process
BP	GO:0043043	1.517E-03	-0.256	45.14	28	260	peptide biosynthetic process
BP	GO:0034645	1.832E-03	-0.139	146.02	118	841	cellular macromolecule biosynthetic process
BP	GO:0043413	1.914E-03	0.442	7.64	16	44	macromolecule glycosylation
BP	GO:0016485	1.979E-03	0.532	5.04	12	29	protein processing
BP	GO:0016558	2.395E-03	0.637	3.30	9	19	protein import into peroxisome matrix
BP	GO:0006487	2.788E-03	0.507	5.21	12	30	protein N-linked glycosylation
BP	GO:0006399	2.839E-03	-0.471	15.45	6	89	tRNA metabolic process
BP	GO:0045333	3.254E-03	0.338	12.33	22	71	cellular respiration
BP	GO:0071702	3.419E-03	0.143	90.42	113	525	organic substance transport
BP	GO:0006099	3.513E-03	0.520	4.69	11	27	tricarboxylic acid cycle
BP	GO:0006879	3.846E-03	0.484	5.38	12	31	cellular iron ion homeostasis
BP	GO:0007031	3.947E-03	0.435	6.73	14	39	peroxisome organization
BP	GO:0034470	4.078E-03	-0.541	11.99	4	68	ncRNA processing
BP	GO:0009101	4.135E-03	0.397	8.16	16	47	glycoprotein biosynthetic process
BP	GO:0034654	4.161E-03	-0.154	93.41	72	538	nucleobase-containing compound biosynthetic process
BP	GO:0042273	4.423E-03	-0.918	6.95	1	40	ribosomal large subunit biogenesis
BP	GO:0010907	5.214E-03	Undef	0.52	3	3	positive regulation of glucose metabolic process
BP	GO:0034406	5.214E-03	Undef	0.52	3	3	cell wall beta-glucan metabolic process
BP	GO:0035948	5.214E-03	Undef	0.52	3	3	positive regulation of gluconeogenesis by positive regulation of transcription from RNA polymerase II promoter
BP	GO:0046579	5.214E-03	Undef	0.52	3	3	positive regulation of Ras protein signal transduction

BP	GO:0061414	5.214E-03	Undef	0.52	3	3	positive regulation of transcription from RNA polymerase II promoter by a nonfermentable carbon source
BP	GO:0006896	5.488E-03	0.557	3.65	9	21	Golgi to vacuole transport
BP	GO:0051649	6.555E-03	0.149	66.67	85	384	establishment of localization in cell
BP	GO:0016125	6.893E-03	Undef	4.51	0	26	sterol metabolic process
BP	GO:0043933	7.196E-03	-0.153	80.74	62	465	macromolecular complex subunit organization
BP	GO:0006364	9.496E-03	-0.561	9.42	3	54	rRNA processing
BP	GO:0046685	9.605E-03	0.681	2.08	6	12	response to arsenic-containing substance
BP	GO:0006490	1.006E-02	0.981	1.04	4	6	oligosaccharide-lipid intermediate biosynthetic process
BP	GO:0072594	1.008E-02	0.203	28.14	40	164	establishment of protein localization to organelle
BP	GO:0006605	1.147E-02	0.246	17.56	27	103	protein targeting
BP	GO:0006891	1.241E-02	0.572	2.78	7	16	intra-Golgi vesicle-mediated transport
BP	GO:0006913	1.246E-02	-0.376	14.76	7	85	nucleocytoplasmic transport
BP	GO:0050657	1.265E-02	-0.436	11.98	5	69	nucleic acid transport
BP	GO:0044710	1.272E-02	0.098	158.17	181	911	single-organism metabolic process
BP	GO:0006400	1.311E-02	-0.541	9.03	3	52	tRNA modification
BP	GO:0016042	1.311E-02	-0.541	9.03	3	52	lipid catabolic process
BP	GO:0016192	1.380E-02	0.163	41.67	55	240	vesicle-mediated transport
BP	GO:0006974	1.397E-02	-0.225	32.12	21	185	cellular response to DNA damage stimulus
BP	GO:0010821	1.526E-02	0.614	2.26	6	13	regulation of mitochondrion organization
BP	GO:0051236	1.622E-02	-0.421	11.63	5	67	establishment of RNA localization
BP	GO:0006091	1.670E-02	0.209	22.22	32	128	generation of precursor metabolites and energy
BP	GO:0043648	1.740E-02	0.529	2.93	7	17	dicarboxylic acid metabolic process
BP	GO:0015865	1.801E-02	0.526	2.95	7	17	purine nucleotide transport
BP	GO:0019725	1.805E-02	0.187	27.43	38	158	cellular homeostasis
BP	GO:0006102	1.815E-02	1.157	0.69	3	4	isocitrate metabolic process
BP	GO:0007050	1.815E-02	1.157	0.69	3	4	cell cycle arrest
BP	GO:0016569	1.844E-02	-0.302	18.06	10	104	chromatin modification
BP	GO:1901576	1.998E-02	-0.087	201.23	179	1159	organic substance biosynthetic process
BP	GO:0006084	2.026E-02	0.805	1.22	4	7	acetyl-CoA metabolic process
BP	GO:0006116	2.026E-02	0.805	1.22	4	7	NADH oxidation
BP	GO:0055076	2.256E-02	0.263	11.98	19	69	transition metal ion homeostasis

BP	GO:0044700	2.323E-02	0.154	38.37	50	221	single organism signaling
BP	GO:0071426	2.345E-02	-0.441	9.72	4	56	ribonucleoprotein complex export from nucleus
BP	GO:0030001	2.461E-02	0.266	11.29	18	65	metal ion transport
BP	GO:0006623	2.495E-02	0.323	7.42	13	43	protein targeting to vacuole
BP	GO:0006915	2.519E-02	0.381	5.21	10	30	apoptotic process
BP	GO:0045184	2.571E-02	0.181	25.47	35	152	establishment of protein localization
BP	GO:0000959	2.643E-02	Undef	3.30	0	19	mitochondrial RNA metabolic process
BP	GO:0006144	2.643E-02	Undef	3.30	0	19	purine nucleobase metabolic process
BP	GO:0042886	2.643E-02	Undef	3.30	0	19	amide transport
BP	GO:0006302	2.661E-02	-0.433	9.55	4	55	double-strand break repair
BP	GO:0010256	2.684E-02	0.270	10.59	17	61	endomembrane system organization
BP	GO:0042274	2.699E-02	-0.776	5.06	1	29	ribosomal small subunit biogenesis
BP	GO:0000398	2.768E-02	-0.773	5.04	1	29	mRNA splicing, via spliceosome
BP	GO:0006323	2.768E-02	-0.773	5.04	1	29	DNA packaging
BP	GO:0034728	2.792E-02	-0.582	6.60	2	38	nucleosome organization
BP	GO:0097033	2.870E-02	0.601	1.91	5	11	mitochondrial respiratory chain complex III biogenesis
BP	GO:0007264	2.925E-02	0.274	9.90	16	57	small GTPase mediated signal transduction
BP	GO:0000751	3.011E-02	Undef	0.35	2	2	mitotic cell cycle arrest in response to pheromone
BP	GO:0006104	3.011E-02	Undef	0.35	2	2	succinyl-CoA metabolic process
BP	GO:0006105	3.011E-02	Undef	0.35	2	2	succinate metabolic process
BP	GO:0006482	3.011E-02	Undef	0.35	2	2	protein demethylation
BP	GO:0009107	3.011E-02	Undef	0.35	2	2	lipoate biosynthetic process
BP	GO:0015700	3.011E-02	Undef	0.35	2	2	arsenite transport
BP	GO:0019858	3.011E-02	Undef	0.35	2	2	cytosine metabolic process
BP	GO:0032298	3.011E-02	Undef	0.35	2	2	positive regulation of DNA-dependent DNA replication initiation
BP	GO:0045046	3.011E-02	Undef	0.35	2	2	protein import into peroxisome membrane
BP	GO:0046688	3.011E-02	Undef	0.35	2	2	response to copper ion
BP	GO:0051187	3.011E-02	Undef	0.35	2	2	cofactor catabolic process
BP	GO:0055091	3.011E-02	Undef	0.35	2	2	phospholipid homeostasis
BP	GO:0061157	3.011E-02	Undef	0.35	2	2	mRNA destabilization
BP	GO:0061415	3.011E-02	Undef	0.35	2	2	negative regulation of transcription from RNA polymerase II promoter by a nonfermentable carbon source

BP	GO:0070880	3.011E-02	Undef	0.35	2	2	fungal-type cell wall beta-glucan biosynthetic process
BP	GO:0097320	3.011E-02	Undef	0.35	2	2	membrane tubulation
BP	GO:0097352	3.011E-02	Undef	0.35	2	2	autophagosome maturation
BP	GO:1901564	3.081E-02	-0.099	113.73	97	655	organonitrogen compound metabolic process
BP	GO:0022618	3.138E-02	-0.572	6.46	2	37	ribonucleoprotein complex assembly
BP	GO:0044764	3.140E-02	0.213	16.49	24	95	multi-organism cellular process
BP	GO:0008219	3.160E-02	0.359	5.38	10	31	cell death
BP	GO:0065002	3.160E-02	0.305	7.64	13	44	intracellular protein transmembrane transport
BP	GO:0051301	3.162E-02	0.175	25.00	34	144	cell division
BP	GO:0048878	3.169E-02	0.168	27.61	37	159	chemical homeostasis
BP	GO:0046496	3.185E-02	0.279	9.20	15	53	nicotinamide nucleotide metabolic process
BP	GO:0000028	3.201E-02	Undef	3.13	0	18	ribosomal small subunit assembly
BP	GO:0000469	3.201E-02	Undef	3.13	0	18	cleavage involved in rRNA processing
BP	GO:0043044	3.201E-02	Undef	3.13	0	18	ATP-dependent chromatin remodeling
BP	GO:0097659	3.210E-02	-0.118	76.05	62	438	nucleic acid-templated transcription
BP	GO:0006997	3.228E-02	-0.569	6.42	2	37	nucleus organization
BP	GO:0006298	3.266E-02	0.504	2.60	6	15	mismatch repair
BP	GO:0015867	3.266E-02	0.504	2.60	6	15	ATP transport
BP	GO:0030242	3.266E-02	0.504	2.60	6	15	pexophagy
BP	GO:0070592	3.266E-02	0.504	2.60	6	15	cell wall polysaccharide biosynthetic process
BP	GO:0051186	3.331E-02	0.182	22.51	31	130	cofactor metabolic process
BP	GO:0006405	3.403E-02	-0.473	7.81	3	45	RNA export from nucleus
BP	GO:0006826	3.404E-02	0.447	3.30	7	19	iron ion transport
BP	GO:0006875	3.449E-02	0.238	12.50	19	72	cellular metal ion homeostasis
BP	GO:0007154	3.475E-02	0.124	51.91	64	299	cell communication
BP	GO:0000917	3.504E-02	0.680	1.39	4	8	barrier septum assembly
BP	GO:0044419	3.504E-02	0.680	1.39	4	8	interspecies interaction between organisms
BP	GO:0045913	3.504E-02	0.680	1.39	4	8	positive regulation of carbohydrate metabolic process
BP	GO:0016052	3.613E-02	0.250	10.94	17	63	carbohydrate catabolic process
BP	GO:0006886	3.676E-02	0.278	8.56	14	51	intracellular protein transport
BP	GO:0051276	3.710E-02	-0.197	27.59	19	158	chromosome organization
BP	GO:0000723	3.740E-02	-0.743	4.71	1	27	telomere maintenance
BP	GO:0090304	3.752E-02	-0.198	27.49	19	150	nucleic acid metabolic process

BP	GO:0044085	3.807E-02	-0.102	94.80	80	546	cellular component biogenesis
BP	GO:0007062	3.823E-02	-0.740	4.69	1	27	sister chromatid cohesion
BP	GO:0000466	3.878E-02	Undef	2.95	0	17	maturation of 5.8S rRNA from tricistronic rRNA transcript (SSU-rRNA, 5.8S rRNA, LSU-rRNA)
BP	GO:0000722	3.878E-02	Undef	2.95	0	17	telomere maintenance via recombination
BP	GO:0031938	3.878E-02	Undef	2.95	0	17	regulation of chromatin silencing at telomere
BP	GO:0046148	3.878E-02	Undef	2.95	0	17	pigment biosynthetic process
BP	GO:0000042	3.955E-02	0.855	0.87	3	5	protein targeting to Golgi
BP	GO:0006828	3.955E-02	0.855	0.87	3	5	manganese ion transport
BP	GO:0090150	3.959E-02	0.253	10.24	16	59	establishment of protein localization to membrane
BP	GO:1901135	4.176E-02	0.141	35.25	45	203	carbohydrate derivative metabolic process
BP	GO:0006348	4.193E-02	-0.546	6.11	2	35	chromatin silencing at telomere
BP	GO:0045934	4.239E-02	-0.168	34.20	25	197	negative regulation of nucleobase-containing compound metabolic process
BP	GO:0016051	4.325E-02	0.230	11.98	18	69	carbohydrate biosynthetic process
BP	GO:0006368	4.360E-02	-0.397	8.85	4	51	transcription elongation from RNA polymerase II promoter
BP	GO:0010038	4.422E-02	0.413	3.46	7	20	response to metal ion
BP	GO:0015748	4.447E-02	0.277	7.99	13	46	organophosphate ester transport
BP	GO:1902580	4.471E-02	0.153	28.30	37	163	single-organism cellular localization
BP	GO:0009967	4.479E-02	0.412	3.47	7	20	positive regulation of signal transduction
BP	GO:0030968	4.479E-02	0.412	3.47	7	20	endoplasmic reticulum unfolded protein response
BP	GO:0006536	4.481E-02	0.458	2.78	6	16	glutamate metabolic process
BP	GO:0000462	4.486E-02	-0.723	4.51	1	26	maturation of SSU-rRNA from tricistronic rRNA transcript (SSU-rRNA, 5.8S rRNA, LSU-rRNA)
BP	GO:0009086	4.486E-02	-0.723	4.51	1	26	methionine biosynthetic process
BP	GO:0043094	4.486E-02	-0.723	4.51	1	26	cellular metabolic compound salvage
BP	GO:0006733	4.565E-02	0.243	10.42	16	60	oxidoreduction coenzyme metabolic process
BP	GO:0006694	4.697E-02	Undef	2.78	0	16	steroid biosynthetic process
BP	GO:0034502	4.697E-02	Undef	2.78	0	16	protein localization to chromosome

BP	GO:0051348	4.697E-02	Undef	2.78	0	16	negative regulation of transferase activity
BP	GO:0006355	4.701E-02	-0.109	73.62	61	424	regulation of transcription, DNA-templated
BP	GO:2001141	4.701E-02	-0.109	73.62	61	424	regulation of RNA biosynthetic process
BP	GO:0032258	4.767E-02	0.319	5.73	10	33	CVT pathway
BP	GO:0000070	4.946E-02	-0.320	11.29	6	65	mitotic sister chromatid segregation
BP	GO:0000375	4.952E-02	-0.530	5.90	2	34	RNA splicing, via transesterification reactions
BP	GO:0006887	4.952E-02	-0.530	5.90	2	34	exocytosis
BP	GO:0006873	4.972E-02	0.180	18.93	26	109	cellular ion homeostasis
BP	GO:0098771	4.972E-02	0.180	18.93	26	109	inorganic ion homeostasis
CC	GO:0044391	3.952E-05	-0.522	25.52	9	147	ribosomal subunit
CC	GO:0043232	5.490E-05	-0.203	122.41	88	705	intracellular non-membrane-bounded organelle
CC	GO:1990904	2.409E-04	-0.268	57.30	35	330	ribonucleoprotein complex
CC	GO:0005794	3.952E-04	0.280	28.65	46	165	Golgi apparatus
CC	GO:0022627	5.638E-04	Undef	6.77	0	39	cytosolic small ribosomal subunit
CC	GO:0098588	6.614E-04	0.184	70.65	95	410	bounding membrane of organelle
CC	GO:0012505	7.475E-04	0.172	82.46	108	489	endomembrane system
CC	GO:0044695	9.017E-04	Undef	0.69	4	4	Dsc E3 ubiquitin ligase complex
CC	GO:0044427	1.008E-03	-0.321	33.34	18	192	chromosomal part
CC	GO:0010008	2.357E-03	0.374	10.59	20	61	endosome membrane
CC	GO:0030125	2.988E-03	0.857	1.74	6	10	clathrin vesicle coat
CC	GO:0030119	3.005E-03	0.748	2.26	7	13	AP-type membrane coat adaptor complex
CC	GO:0005654	3.787E-03	-0.386	19.27	9	111	nucleoplasm
CC	GO:0030121	3.885E-03	1.282	0.87	4	5	AP-1 adaptor complex
CC	GO:0030687	4.693E-03	Undef	4.86	0	28	preribosome, large subunit precursor
CC	GO:0098589	6.226E-03	0.506	4.34	10	25	membrane region
CC	GO:0044425	6.520E-03	0.112	149.45	174	886	membrane part
CC	GO:0070603	6.893E-03	Undef	4.51	0	26	SWI/SNF superfamily-type complex
CC	GO:0098805	7.812E-03	0.151	60.81	78	355	whole membrane
CC	GO:0031410	8.013E-03	0.295	13.20	22	76	cytoplasmic vesicle
CC	GO:0031988	8.013E-03	0.295	13.20	22	76	membrane-bounded vesicle
CC	GO:0031307	8.185E-03	0.623	2.60	7	15	integral component of mitochondrial outer membrane
CC	GO:1990204	8.419E-03	0.479	4.50	10	26	oxidoreductase complex
CC	GO:0030136	8.937E-03	0.445	5.21	11	30	clathrin-coated vesicle
CC	GO:0005737	9.089E-03	0.112	459.90	483	2687	cytoplasm
CC	GO:0030662	9.937E-03	0.543	3.30	8	19	coated vesicle membrane

CC	GO:0012510	1.006E-02	0.981	1.04	4	6	trans-Golgi network transport vesicle membrane
CC	GO:1902495	1.060E-02	0.777	1.56	5	9	transmembrane transporter complex
CC	GO:0000228	1.088E-02	-0.257	27.78	17	160	nuclear chromosome
CC	GO:0005777	1.189E-02	0.300	11.29	19	65	peroxisome
CC	GO:0048475	1.410E-02	0.505	3.47	8	20	coated membrane
CC	GO:0012506	1.526E-02	0.427	4.86	10	28	vesicle membrane
CC	GO:0005770	1.767E-02	0.349	7.12	13	41	late endosome
CC	GO:0017119	1.815E-02	1.157	0.69	3	4	Golgi transport complex
CC	GO:0005778	1.977E-02	0.403	5.04	10	29	peroxisomal membrane
CC	GO:1990429	2.516E-02	0.485	3.13	7	18	peroxisomal importomer complex
CC	GO:0005789	2.553E-02	0.154	36.81	48	212	endoplasmic reticulum membrane
CC	GO:1902554	2.643E-02	Undef	3.30	0	19	serine/threonine protein kinase complex
CC	GO:0000322	2.658E-02	0.124	59.38	73	342	storage vacuole
CC	GO:0000323	2.658E-02	0.124	59.38	73	342	lytic vacuole
CC	GO:0022625	2.661E-02	-0.433	9.55	4	55	cytosolic large ribosomal subunit
CC	GO:0030062	2.995E-02	Undef	0.35	2	2	mitochondrial tricarboxylic acid cycle enzyme complex
CC	GO:0044432	2.995E-02	0.515	2.56	6	15	endoplasmic reticulum part
CC	GO:0005962	3.011E-02	Undef	0.35	2	2	mitochondrial isocitrate dehydrogenase complex (NAD+)
CC	GO:0009353	3.011E-02	Undef	0.35	2	2	mitochondrial oxoglutarate dehydrogenase complex
CC	GO:0043291	3.011E-02	Undef	0.35	2	2	RAVE complex
CC	GO:0045240	3.011E-02	Undef	0.35	2	2	dihydrolipoyl dehydrogenase complex
CC	GO:0097196	3.011E-02	Undef	0.35	2	2	Shu complex
CC	GO:1990531	3.011E-02	Undef	0.35	2	2	Lem3p-Dnf1p complex
CC	GO:0031974	3.029E-02	-0.108	95.67	80	551	membrane-enclosed lumen
CC	GO:0070013	3.029E-02	-0.108	95.67	80	551	intracellular organelle lumen
CC	GO:0000781	3.228E-02	-0.569	6.42	2	37	chromosome, telomeric region
CC	GO:0016021	3.499E-02	0.080	157.48	176	912	integral component of membrane
CC	GO:0000328	3.504E-02	0.680	1.39	4	8	fungal-type vacuole lumen
CC	GO:0005750	3.504E-02	0.680	1.39	4	8	mitochondrial respiratory chain complex III
CC	GO:0043596	3.878E-02	Undef	2.95	0	17	nuclear replication fork
CC	GO:0005798	4.345E-02	0.380	4.17	8	24	Golgi-associated vesicle
CC	GO:0061695	4.443E-02	-0.327	11.46	6	66	transferase complex, transferring phosphorus-containing groups
CC	GO:0044437	4.529E-02	0.124	44.45	55	256	vacuolar part

CC	GO:0044428	4.559E-02	-0.155	38.50	29	215	nuclear part
CC	GO:0008023	4.697E-02	Undef	2.78	0	16	transcription elongation factor complex
CC	GO:0000790	4.725E-02	-0.282	13.89	8	80	nuclear chromatin
CC	GO:0005618	4.776E-02	0.200	15.45	22	89	cell wall
MF	GO:0044822	1.080E-04	Undef	8.25	0	47	poly(A) RNA binding
MF	GO:0005199	1.187E-04	0.938	2.43	9	14	structural constituent of cell wall
MF	GO:0003735	1.508E-04	-0.488	23.79	9	137	structural constituent of ribosome
MF	GO:0003676	5.664E-04	-0.180	106.61	79	614	nucleic acid binding
MF	GO:0030276	7.974E-04	0.925	1.91	7	11	clathrin binding
MF	GO:0019843	3.872E-03	Undef	5.04	0	29	rRNA binding
MF	GO:0016417	1.006E-02	0.981	1.04	4	6	S-acyltransferase activity
MF	GO:0016796	2.643E-02	Undef	3.30	0	19	exonuclease activity, active with either ribo- or deoxyribonucleic acids and producing 5'-phosphomonoesters
MF	GO:0004725	2.870E-02	0.601	1.91	5	11	protein tyrosine phosphatase activity
MF	GO:0004860	2.870E-02	0.601	1.91	5	11	protein kinase inhibitor activity
MF	GO:0016722	2.870E-02	0.601	1.91	5	11	oxidoreductase activity, oxidizing metal ions
MF	GO:0004335	3.011E-02	Undef	0.35	2	2	galactokinase activity
MF	GO:0004438	3.011E-02	Undef	0.35	2	2	phosphatidylinositol-3-phosphatase activity
MF	GO:0004449	3.011E-02	Undef	0.35	2	2	isocitrate dehydrogenase (NAD+) activity
MF	GO:0004775	3.011E-02	Undef	0.35	2	2	succinate-CoA ligase (ADP-forming) activity
MF	GO:0004862	3.011E-02	Undef	0.35	2	2	cAMP-dependent protein kinase inhibitor activity
MF	GO:0005227	3.011E-02	Undef	0.35	2	2	calcium activated cation channel activity
MF	GO:0016405	3.011E-02	Undef	0.35	2	2	CoA-ligase activity
MF	GO:0016624	3.011E-02	Undef	0.35	2	2	oxidoreductase activity, acting on the aldehyde or oxo group of donors, disulfide as acceptor
MF	GO:0016979	3.011E-02	Undef	0.35	2	2	lipoate-protein ligase activity
MF	GO:0032794	3.011E-02	Undef	0.35	2	2	GTPase activating protein binding
MF	GO:0042281	3.011E-02	Undef	0.35	2	2	dolichyl pyrophosphate Man9GlcNAc2 alpha-1,3-glucosyltransferase activity
MF	GO:0042392	3.011E-02	Undef	0.35	2	2	sphingosine-1-phosphate phosphatase activity
MF	GO:0070569	3.011E-02	Undef	0.35	2	2	uridylyltransferase activity
MF	GO:0031406	3.201E-02	Undef	3.13	0	18	carboxylic acid binding
MF	GO:0001076	3.228E-02	-0.569	6.42	2	37	transcription factor activity, RNA polymerase

							II transcription factor binding
MF	GO:0042578	3.793E-02	0.213	15.11	22	87	phosphoric ester hydrolase activity
MF	GO:0003756	3.955E-02	0.855	0.87	3	5	protein disulfide isomerase activity
MF	GO:0004707	3.955E-02	0.855	0.87	3	5	MAP kinase activity
MF	GO:0004713	3.955E-02	0.855	0.87	3	5	protein tyrosine kinase activity
MF	GO:0005381	4.234E-02	0.534	2.08	5	12	iron ion transmembrane transporter activity
MF	GO:0005507	4.234E-02	0.534	2.08	5	12	copper ion binding
MF	GO:0003729	4.399E-02	-0.257	16.49	10	95	mRNA binding
